# Release of Ku and MRN from DNA Ends by Mre11 Nuclease Activity and Ctp1 Is Required for Homologous Recombination Repair of Double-Strand Breaks

**DOI:** 10.1371/journal.pgen.1002271

**Published:** 2011-09-08

**Authors:** Petra Langerak, Eva Mejia-Ramirez, Oliver Limbo, Paul Russell

**Affiliations:** Department of Molecular Biology, The Scripps Research Institute, La Jolla, California, United States of America; The University of North Carolina at Chapel Hill, United States of America

## Abstract

The multifunctional Mre11-Rad50-Nbs1 (MRN) protein complex recruits ATM/Tel1 checkpoint kinase and CtIP/Ctp1 homologous recombination (HR) repair factor to double-strand breaks (DSBs). HR repair commences with the 5′-to-3′ resection of DNA ends, generating 3′ single-strand DNA (ssDNA) overhangs that bind Replication Protein A (RPA) complex, followed by Rad51 recombinase. In *Saccharomyces cerevisiae*, the Mre11-Rad50-Xrs2 (MRX) complex is critical for DSB resection, although the enigmatic ssDNA endonuclease activity of Mre11 and the DNA-end processing factor Sae2 (CtIP/Ctp1 ortholog) are largely unnecessary unless the resection activities of Exo1 and Sgs1-Dna2 are also eliminated. Mre11 nuclease activity and Ctp1/CtIP are essential for DSB repair in *Schizosaccharomyces pombe* and mammals. To investigate DNA end resection in *Schizo. pombe*, we adapted an assay that directly measures ssDNA formation at a defined DSB. We found that Mre11 and Ctp1 are essential for the efficient initiation of resection, consistent with their equally crucial roles in DSB repair. Exo1 is largely responsible for extended resection up to 3.1 kb from a DSB, with an activity dependent on Rqh1 (Sgs1) DNA helicase having a minor role. Despite its critical function in DSB repair, Mre11 nuclease activity is not required for resection in fission yeast. However, Mre11 nuclease and Ctp1 are required to disassociate the MRN complex and the Ku70-Ku80 nonhomologous end-joining (NHEJ) complex from DSBs, which is required for efficient RPA localization. Eliminating Ku makes Mre11 nuclease activity dispensable for MRN disassociation and RPA localization, while improving repair of a one-ended DSB formed by replication fork collapse. From these data we propose that release of the MRN complex and Ku from DNA ends by Mre11 nuclease activity and Ctp1 is a critical step required to expose ssDNA for RPA localization and ensuing HR repair.

## Author Summary

A double-strand break (DSB) is a devastating form of DNA damage. Fortunately, cells are equipped with two DSB repair pathways: homologous recombination (HR) and nonhomologous end-joining (NHEJ). The Mre11-Rad50-Nbs1 (MRN) protein complex recognizes DSBs and initiates HR repair. The Mre11 subunit harbors a nuclease domain that is essential for repair in fission yeast and mammals, although the function is unknown. Here we show that Mre11 nuclease activity is required to release the Ku complex from DNA ends in fission yeast. While the initiation of repair, i.e. the generation of single-stranded DNA (ssDNA) overhangs in Mre11-nuclease dead mutants, is unaffected, we find that an essential downstream step involving the localization of Replication Protein A (RPA) to ssDNA is substantially decreased due to the inability to release Ku and MRN from the DNA end. In contrast, a DNA processing factor called Ctp1, which binds to Nbs1, is essential for the initiation of repair as well as the release of Ku and MRN from DNA ends. Importantly, we find that efficient localization of RPA, which is essential for efficient DSB repair by HR, requires the release of Ku and MRN from the DNA by the combined action of Mre11 nuclease and Ctp1.

## Introduction

Genome integrity is constantly threatened by DNA damage resulting from internal and external insults. One of the most harmful forms of DNA damage is the double-strand break (DSB). When left unrepaired, DSBs can cause a plethora of chromosomal aberrations that often result in cell death or mutations that can lead to cancer phenotypes [1]. There are two major DSB repair pathways: nonhomologous end-joining (NHEJ) and homologous recombination (HR). NHEJ is an error-prone pathway that repairs DSBs by directly ligating DNA ends with little or no end processing. Key NHEJ proteins that are conserved from yeast to humans include the Ku70-Ku80 heterodimer, which binds DNA ends with high affinity, as well as XLF/Cernunnos and DNA ligase IV [2]–[5]. HR typically uses the intact sister chromatid as template for repair in mitotic cells, hence it is most critical during S and G2 phases of the cell cycle [6], [7]. HR is also essential for recombination between homologous chromosomes in meiosis, which is required for proper chromosome segregation and generation of genetic diversity. A multitude of human disease syndromes have been traced to NHEJ and HR defects, including those characterized by neurological, immunological and developmental disorders as well as radiation sensitivity, premature aging diseases and cancer, pointing to the critical roles of NHEJ and HR in maintaining genome stability [8]–[13].

A major prerequisite for HR is 5′ to 3′ resection of the DNA flanking each side of the break [14], [15]. The eukaryotic heterotrimeric Replication Protein A (RPA) binds the single-stranded DNA (ssDNA). The recruitment of the ATR (ataxia-telangiectasia mutated- and Rad3-related)-ATRIP (ATR-interacting protein) protein kinase complex to RPA-coated ssDNA initiates checkpoint signaling [16]. RPA is exchanged for Rad51, a protein involved in the search for homology to allow subsequent repair [15]. Research in multiple model organisms has shed light on the proteins that catalyze the initiating events of HR, with most of the groundbreaking studies performed in *Saccharomyces cerevisiae*. Current models propose that the Mre11-Rad50-Nbs1 (MRN) protein complex initially recognizes the break, whereupon it initiates HR repair and a checkpoint response through ATM checkpoint kinase. Several years ago, we described a protein in *Schizosaccharomyces pombe* known as Ctp1 (or Nip1) that collaborates with the MRN complex in HR [17]–[19]. Ctp1 shares a conserved C-terminal domain with *S. cerevisiae* Sae2 and mammalian CtIP, the latter of which associates with the MRN complex and the tumor suppressor BRCA1 [20]–[23]. Biochemical studies of Sae2 (also known as Com1) identified a nuclease activity [24], and more recent studies in budding yeast implicated both Sae2 and the Mre11 complex in the initiation of resection required for HR [25]–[31]. Further resection, extending several kilo-base pairs or beyond, is performed by the exonuclease Exo1, or the helicase Sgs1 (Rqh1 in *Schizo. pombe* and BLM in mammals) together with the exonuclease Dna2. Recently, DNA end resection was reconstituted *in vitro* using the budding yeast Mre11 complex, Sgs1-Top3-Rmi1, Dna2 and RPA or the Mre11 complex, Sae2 and Exo1 respectively [32]–[34]. So far, quantitative measurements of the formation of ssDNA by resection of DNA ends have only been performed in *S. cerevisiae*. Studies on the *in vivo* function of orthologous proteins in other organisms have mainly relied upon the indirect detection of ssDNA through the recruitment of RPA to sites of DNA damage. The observation that siRNA depletion of Exo1, BLM and CtIP reduces RPA interactions with damaged DNA in human cells implies that the function of these proteins in resection is conserved from yeast to human [23], [35], [36].

Whilst budding yeast Sae2 can act with the Mre11 complex to initiate resection of a DSB [27]–[29], and it is essential for processing meiotic DSBs created by Spo11 [37]–[40], Sae2 is not generally required for DSB repair. Indeed, *sae2Δ* mutants have a normal growth rate and are only weakly radiosensitive [39], [41]. *Schizo. pombe ctp1Δ* mutants are also unable to process meiotic DSBs created by the Spo11 ortholog Rec12 [42]–[44], but in addition they are acutely sensitive to IR and other DNA break inducing agents [19]. This damage sensitivity is reflected in vertebrate cells with the use of CtIP siRNA knockdown [23], [45]. DNA damage sensitivity is also observed in mutants for the conserved ssDNA endonuclease and 3′ to 5′ dsDNA exonuclease activities of Mre11 (also known as Rad32 in fission yeast). The *mre11-H134S* mutation (corresponding to the *Pyrococcus furiosus mre11-H85S* mutation [46] that ablates both nucleolytic activities) causes severe radiosensitivity in fission yeast, whereas a mutation that specifically impairs exonuclease activity has little effect, indicating that the endonuclease activity is critical for DSB repair in *Schizo. pombe* [46]. Mutations equivalent to *mre11-H134S* in budding yeast are mildly IR sensitive but do not impair DSB resection [47], [48], whilst a corresponding mutation of mouse Mre11 causes early embryonic lethality and strong cellular radiosensitivity [49]. Thus, while Mre11 nuclease activity and Ctp1/CtIP are critical for mitotic DSB repair in fission yeast and mice, the underlying molecular defects are largely unknown.

To uncover the critical functions of Mre11 nuclease activity and Ctp1 in *Schizo. pombe* and study the interplay between proteins involved in resection and the localization of RPA to ssDNA overhangs, we have adapted a qPCR-based assay that directly measures the formation of ssDNA at a site-specific DSB. In agreement with studies of Mre11 in budding yeast [27], [28], [50], this assay reveals that Mre11, but not its nuclease activity *per se*, is critical for resection in fission yeast. Ctp1 and Mre11 are both critical for resection in fission yeast, unlike the relationship between Sae2 and Mre11 in budding yeast [25]–[31]. In agreement with studies of Exo1 and Sgs1 in budding yeast [27], [28], Exo1 and Rqh1 comprise independent activities that can catalyze extended resection in fission yeast, although our studies show that Exo1 plays a more dominant role in fission yeast. Also consistent with studies in budding yeast [26], chromatin immunoprecipitation studies indicate that Ctp1 and Mre11 nuclease activity promote the release Ku from DNA ends in fission yeast. The inability to release Ku stabilizes binding of MRN to the break. Intriguingly, despite normal levels of ssDNA, we show that RPA localization in the Mre11 nuclease mutant is reduced compared to wild type by chromatin immunoprecipitation at a site-specific DSB. The observed reduction in RPA localization appears to be a general defect of *mre11-H134S* cells, which is independent of the DSB inducing agent, as IR treatment of these mutant cells results in reduced phosphorylation of the checkpoint kinase Chk1 as compared to wild type. Deletion of Ku in *mre11-H134S* cells reduces MRN retention at the DSB, increases the localization of RPA, improves checkpoint signaling in response to IR, and suppresses sensitivity to DNA damage. From these data we propose that Mre11 nuclease activity and Ctp1 are required to release both the MRN and Ku complexes from DNA ends, which is critical for correct assembly of RPA on the resected DNA ends and the repair of DSBs.

## Results

### A DSB resection assay in fission yeast

**Figure 1:**
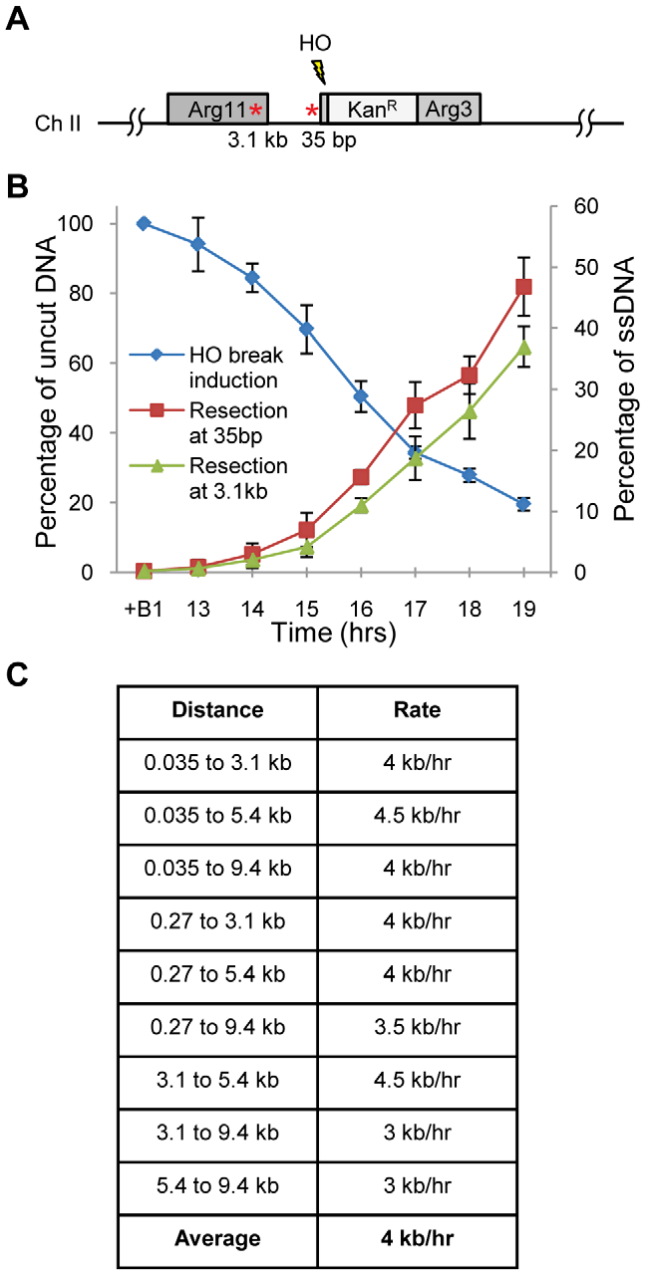
Set-up of resection assay in *Schizo. pombe*. A. Schematic representation of the HO recognition site (⚡) and *ApoI* restriction sites (*) on chromosome two in *Schizo. pombe*. *ApoI* digestion of the DNA cuts dsDNA, but leaves ssDNA intact. B. Typical HO endonuclease induction in wild type is shown. Left vertical axis shows appearance of the break, while the right vertical axis depicts the percentage of ssDNA as a measure for resection. “+B1” represents the repressed condition (HO endonuclease off). Derepression of the HO gene and the appearance of the HO break starts ∼13 hours after induction and continues for several hours. Average and standard deviation (error bar) of three independent experiments are shown. C. Resection rates for wild type are estimated by measuring resection at different distances from the break.

Repair of DSBs by homologous recombination requires 5′-to-3′ resection of DNA ends at each side of the DSB. In budding yeast, DNA end resection assays have been established that quantitatively measure the formation of ssDNA at particular distances from a site-specific DSB. To extend quantitative DSB resection studies to another model organism, in this case *Schizo. pombe*, we adapted a qPCR-based resection assay that measures the formation of ssDNA directly [51]. In this assay, a site-specific DSB is created by the *S. cerevisiae* HO endonuclease, which is expressed from the thiamine-repressible *nmt41* promoter in *Schizo. pombe* [52], [53]. The HO recognition site is integrated at unique sequences at the *arg3* locus, hence these DSBs are irreparable when both sister chromatids are cleaved at this site. The *nmt41* promoter system allows essentially complete repression of the HO expression in the presence of thiamine, which for this assay was a critical attribute of *nmt41* in comparison to other promoters used for regulating gene expression in fission yeast. A disadvantage of the *nmt41* promoter is that full derepression requires ∼16 hrs growth in thiamine-free media, making it impractical to use cell cycle synchronization protocols. However, as *Schizo. pombe* has a very short G1 phase and a long G2 phase [54], and HO-induced DSBs elicit a robust checkpoint response that arrests division in late G2 phase [52], [53], the large majority of cells (∼80%) in wild type cultures are expected to suffer the HO-induced DSB in G2 phase and remain arrested in this phase of the cell cycle. DSBs do not delay progression through G1 or S phase in fission yeast [55], thus the small fraction of cells that suffer a DSB outside of G2 phase will replicate the DSB once and then arrest in G2. However, as discussed below, resection is required for robust checkpoint signaling. We use qPCR to measure the formation of the break through the corresponding disappearance of the DNA product formed by PCR across the DSB, and calculate the percentage of uncut DNA by comparing these values to the formation of a DNA product elsewhere in the genome. These data are used to correct for timing variations in break formation between strains and experiments. Data were generated from a minimum of 3 independent experiments. Resection initiation was measured 35 bp from the break, whereas extended resection was measured 3.1 kb from the DSB (described in Materials and Methods, see Figure 1A, Figure S1A and S1B). As expected, formation of the break was followed by resection initiation measured at 35 bp, which preceded the appearance of the long-range resection product at 3.1 kb (Figure 1B). By measuring the formation of ssDNA at several sites from 35 bp to 9.4 kb from the break, we estimated that the resection rate was fairly constant in this region, with an average rate of ∼4 kb/hr in wild type (see Figure 1C). Remarkably similar rates (∼3.5–4.5 kb/hr) were measured using a Southern hybridization-based assay in budding yeast [28].

### Ctp1 and Mre11 are equally critical for resection initiation in *Schizo. pombe*

**Figure 2:**
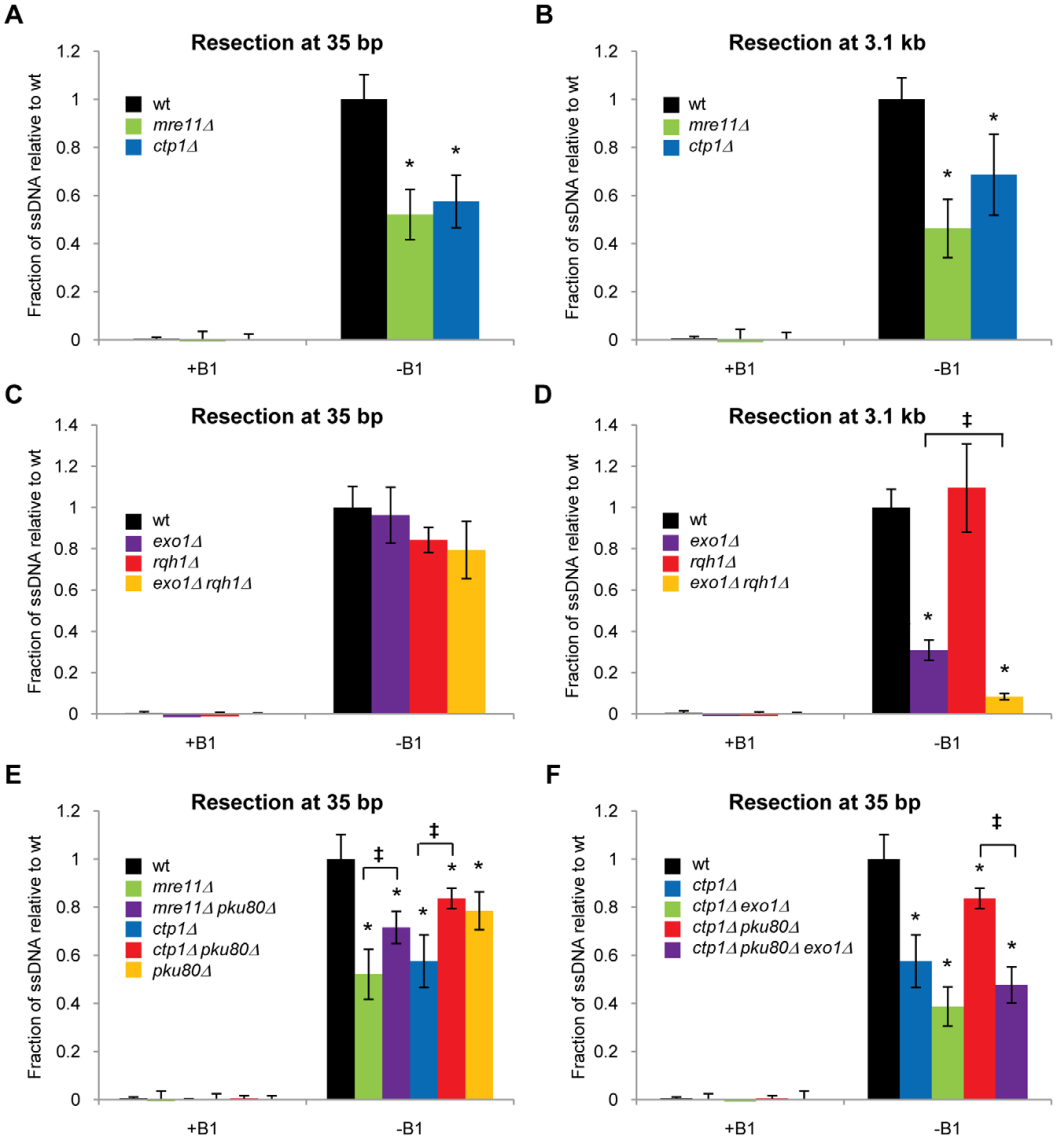
Resection requires Ctp1, Mre11, and Exo1, but not Rqh1, and can be inhibited by Ku. +B1: HO endonuclease expression repressed. −B1: expression of HO endonuclease induced. The percentage of ssDNA in the absence of B1 is shown for the time point at which the amount of cut DNA is maximal, seven hours after the first cells show the appearance of the break. A. Resection at 35 bp from the break is decreased in the absence of either Mre11 or Ctp1. B. Resection at 3.1 kb from the break is decreased in the absence of either Mre11 or Ctp1. C. Resection at 35 bp is minimally affected by deletion of Exo1 and/or Rqh1. D. Resection at 3.1 kb is severely affected in the absence of Exo1, but not Rqh1. An *exo1Δ rqh1Δ* mutant shows a more severe phenotype than *exo1Δ* mutant. E. Resection at 35 bp in the *mre11Δ* and *ctp1Δ* mutants is statistically increased by deletion of Ku (two-tailed Student T-test p-values 0.03 and 0.01, respectively). Resection initiation in the *pku80Δ* strain is significantly reduced relative to wild type. F. Resection initiation in a *ctp1Δ pku80Δ* mutant is significantly decreased by deleting Exo1. Average and standard deviation (error bar) from at least three independent experiments are shown. All values for mutant strains are depicted relative to the average value of induced wild type (−B1). The wild type average was set to 1, with its standard deviation and values for the mutants adjusted by the same factor. Raw data and kinetics are shown in Figure S6. Asterisk depicts statistically significant differences with wild type as determined by a two-tailed Student T-test, p-value≤0.05. ‡ depicts statistically significant differences amongst the bracketed strains as determined by a two-tailed Student T-test, p-value≤0.05.

In *S. cerevisiae*, the Mre11-Rad50-Xrs2^Nbs1^ (MRX) subunits are essential for radioresistance, whereas a role for Sae2 is revealed only at very high doses of IR, and even then the *sae2Δ* phenotype is mild compared to *mrxΔ* mutants [6], [39], [41]. These differences are reflected in resection assays: resection initiation at an HO-induced DSB is delayed but nearly 100% efficient in *sae2Δ* cells, as compared to ∼40% resection initiation deficiency in *mre11Δ* or *rad50Δ* mutants [27], [28]. A requirement for Sae2 is only revealed when Exo1 and Sgs1-dependent resection activities are also eliminated. As Ctp1 and the MRN subunits are both essential for radioresistance in *Schizo. pombe* [19], we tested whether Ctp1 is required for resection initiation and whether its activity is as important as Mre11. We first examined *mre11Δ* cells, wherein we found a substantial resection defect, with the formation of ssDNA at 35 bp from the DSB being ∼45% of the level measured in wild type (Figure 2A). The resection initiation defects of *Schizo. pombe* and *S. cerevisiae mre11Δ* mutants appear to be quite similar, which is consistent with their acute radiosensitive phenotypes. *Schizo. pombe ctp1Δ* cells displayed a strong resection initiation defect that was very similar to *mre11Δ* cells (Figure 2A). They also displayed similar defects when resection was measured at 3.1 kb from the DSB (Figure 2B). Thus, Ctp1 and Mre11 are equally critical for resection in fission yeast, which is consistent with their essential roles in IR survival.

Activation of the checkpoint kinase Chk1 is partially impaired in *mre11Δ* and *ctp1Δ* cells exposed to IR [56], [57], which is consistent with a resection defect. However, the IR-induced G2 checkpoint delay is lengthened in these cells, which is likely caused by their inability to complete DSB repair and relieve the checkpoint signal [56], [57]. These mutants also arrested division in response to the HO-induced DSB, although the HO induction kinetics prevented quantitative assessment of the duration of the checkpoint arrest. Therefore, as seen in budding yeast [25], [28], in our experiments more cells may have leaked through the checkpoint arrest in the resection-defective mutants. In budding yeast, replication increases resection at a DSB, but it is unknown which activities are responsible for this effect [51].

### Exo1 is critical for extended resection in fission yeast

In *S. cerevisiae*, Exo1 and an Sgs1/Dna2-dependent activity catalyze extended resection [27], [28]. The Sgs1-dependent activity is critical for very long-range resection (∼10 kb and longer), but either activity is sufficient for resection to ∼3 kb from a DSB. We examined the roles of Exo1 and Rqh1 (Sgs1) in resection at 35 bp and 3.1 kb from the DSB in fission yeast. Elimination of Exo1 or Rqh1, either alone or in combination, did not decrease ssDNA formation at 35 bp by a statistically significant amount (Figure 2C). In comparison, a small but statistically significant defect in resection initiation was observed in *S. cerevisiae exo1Δ sgs1Δ* cells [28]. When we measured resection at 3.1 kb from the DSB, we found that elimination of Exo1 caused a very strong defect, reducing resection efficiency to about 30% compared to wild type, whereas there was no measurable resection deficiency in *rqh1Δ* cells (Figure 2D). Analysis of the *exo1Δ rqh1Δ* strain revealed that essentially all of the remaining long-range resection activity in *exo1Δ* cells was attributable to Rqh1 (Figure 2D). This result suggests that Rqh1 can contribute to long-range resection in fission yeast, but its activity is relatively inefficient. Thus, while Exo1 is not required for efficient resection initiation, it is responsible for the majority of the long-range resection in fission yeast. This situation contrasts somewhat with budding yeast, in which either Exo1 or the Sgs1-dependent activity is sufficient for extended resection.

### Ku blocks Exo1 from initiating resection of a DSB

Intriguingly, deleting Ku subunits substantially suppress *mrnΔ* and *ctp1Δ* phenotypes in *Schizo. pombe* [19], [46], [58]. While Exo1 is not required for survival of IR or CPT exposure, it is crucial for resistance to DSB inducing agents in a *ctp1Δ pku80Δ* background [19]. One possible mechanism to explain this genetic result is that eliminating Ku allows Exo1 to substitute for Ctp1 to initiate resection. To test whether Ku inhibits Exo1-dependent initiation of resection, we performed resection assays in *pku80Δ* backgrounds. Unexpectedly, we detected a small but statistically significant decrease in the formation of ssDNA at 35 bp in the *pku80Δ* strain (Figure 2E). The physiological significance of this defect is unclear, as *pku80Δ* mutants appear to be fully proficient at HR repair of DSBs [5]. Although deletion of Ctp1 or Mre11 caused a large decrease in close-in resection in a *pku80* ^+^ background (Figure 2A), deleting these genes did not have statistically significant effects in the *pku80Δ* background (Figure 2E). These results suggested that another activity could substitute for Ctp1 and Mre11 in a *pku80Δ* background. In support of this model, deleting Ku significantly increased resection at 35 bp in the *ctp1Δ* and *mre11Δ* backgrounds (Figure 2E, p-values≤0.05). Importantly, this effect entirely depended on Exo1, i.e., deleting Ku did not cause a statistically significant increase in resection in the *ctp1Δ exo1Δ* background (Figure 2F). From these results we conclude that Ku inhibits Exo1 from initiating resection in *ctp1Δ* cells. Analogous results have been reported in budding yeast, where it was found that deleting Ku suppressed the resection delay in *sae2Δ* cells and improved resection in an Exo1-dependent manner in a *rad50Δ* mutant [6], [26].

These data further indicated that there is at least one additional resection activity in *ctp1Δ exo1Δ* double mutant cells, with the most likely candidate being an Rqh1-dependent activity. We attempted to answer this question by creating a *ctp1Δ exo1Δ rqh1Δ* strain, but tetrad analysis revealed that the triple mutant is not viable (Figure S2). The same lethality occurs when *exo1Δ rqh1Δ* is combined with either *mre11Δ* or the *mre11-H134S* nuclease defective allele (Figure S2). This genetic interaction was also observed in *S. cerevisiae*, although in some strain backgrounds *rad50Δ* or *sae2Δ* are not lethal when combined with *exo1Δ sgs1Δ* [27], [28]. Even though we were able to construct *ctp1Δ rqh1Δ* and *mre11Δ rqh1Δ* strains, these double mutants were extremely sick and therefore unsuitable for resection assays.

### Deleting Ku restores RPA localization at a DSB in *ctp1Δ* cells

**Figure 3:**
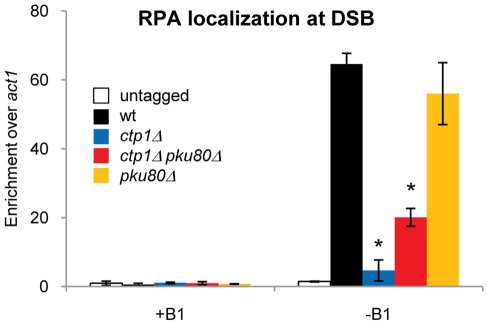
Localization of RPA in *ctp1Δ* mutants is increased in absence of Ku. +B1: HO endonuclease expression repressed. −B1: expression of HO endonuclease induced. Chromatin immunoprecipitation (ChIP) analysis of RPA (rad11-TAP) at 0.2 kb from an HO endonuclease induced DSB. The defective localization of RPA in *ctp1Δ* mutants can be partially restored by deletion of Ku. Average and standard deviation (error bar) of three independent experiments are shown. Asterisk depicts statistically significant differences with wild type as determined by a two-tailed Student T-test, p-value≤0.05.

We have previously established that the localization of RPA to ssDNA is reduced in the absence of Ctp1 or Mre11 [19]. As Ku deletion improves the growth and DNA damage survival of *ctp1Δ* mutants [19], [46], and significantly increases resection initiation performed by Exo1 in the absence of Ctp1 (Figure 2F), we investigated whether Ku deletion also enhances RPA localization at a DSB in *ctp1Δ* cells. We used a previously established chromatin immunoprecipitation (ChIP) protocol to measure RPA enrichment at the HO-induced DSB. The ChIP procedure detects RPA associated with a 200 bp region that overlaps ∼85% with the region used to measure resection at 35 bp from the break, which allows us to directly compare the initiation of resection with protein enrichment. Using RPA ChIP, we confirmed our earlier studies showing a severe reduction in the localization of RPA to the DSB in *ctp1Δ* cells [19], and furthermore found that RPA localization was significantly increased in *ctp1Δ pku80Δ* cells compared to the *ctp1Δ* control, although not to the wild type level (Figure 3). Thus, deleting Ku does not completely restore RPA localization at a DSB in *ctp1Δ* cells, which is consistent with the failure of *pku80Δ* to fully suppress *ctp1Δ* at higher doses of DNA damage [19], [46]. Very similar genetic interactions and effects on RPA localization were observed when Ku is deleted in *mre11Δ* cells (data not shown).

### Ku is enriched at DNA ends in the absence of Mre11 or Ctp1

**Figure 4:**
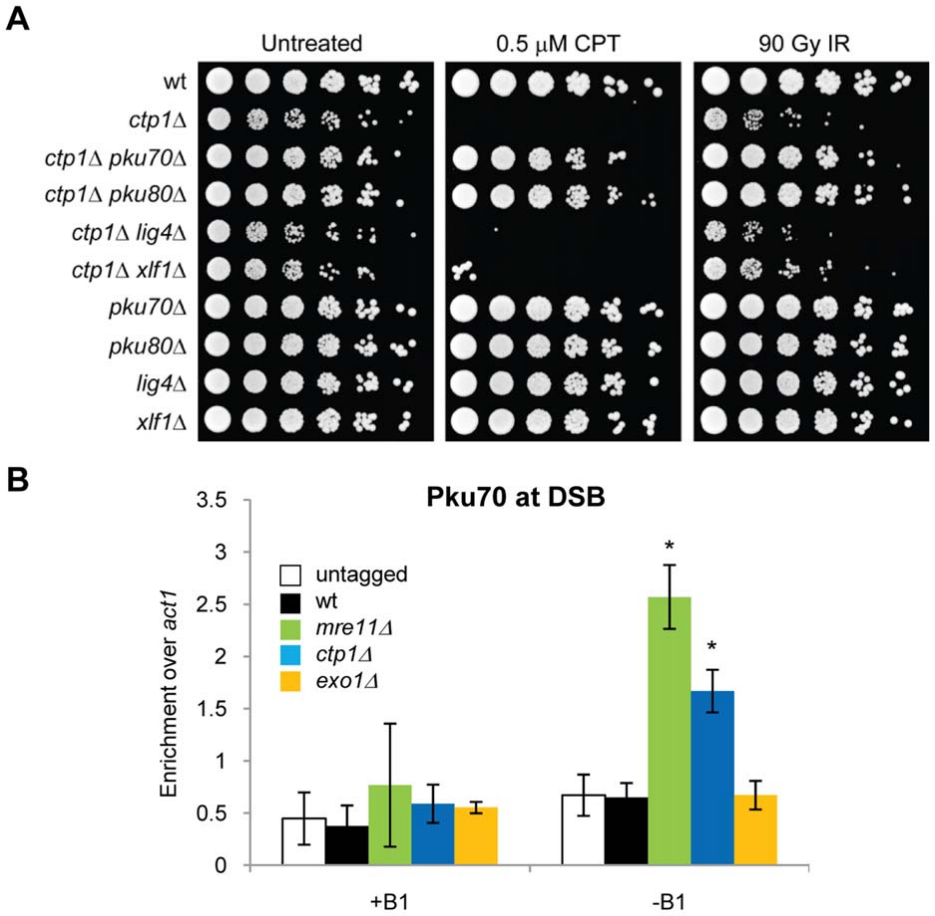
Mre11 and Ctp1 are required to release Ku from DNA ends. A. Dilution assay depicting the rescue effect of the slow growth and DSB sensitivity (induced by IR and CPT) phenotypes of *ctp1Δ* mutant by deletion of Pku70 or Pku80. Deletion of two other NHEJ proteins, Xlf1 and Lig4, does not show the same effect. B. +B1: HO endonuclease expression repressed. −B1: expression of HO endonuclease induced. ChIP analysis of Ku (Pku70-HA) at 0.2 kb from HO-induced DSB. In the absence of Mre11 or Ctp1, the high affinity DNA binding Ku heterodimer is enriched at the HO break compared to wild type. No increase in Ku is observed in wild type compared to an untagged control. Average and standard deviation (error bar) of three independent experiments are shown. Asterisk depicts statistically significant differences with wild type as determined by a two-tailed Student T-test, p-value≤0.05. ‡ depicts statistically significant differences amongst the bracketed strains as determined by a two-tailed Student T-test, p-value≤0.05.

Having found that *pku80Δ* suppression of *ctp1Δ* DNA damage sensitivity correlates with increased DSB resection and RPA localization at a DSB, we investigated whether other mutations that impair NHEJ would suppress *ctp1Δ*. We found that deleting Lig4 or Xlf1 did not rescue the slow growth and genotoxin sensitivity of *ctp1Δ* cells, indicating that it is the DSB-binding capacity of Ku, as opposed to NHEJ *per se*, that blocks Exo1-dependent repair of DSBs in *ctp1Δ* cells (Figure 4A). From these data we hypothesized that Ctp1 might be required to release Ku or to prevent it from binding DNA ends. To test this idea, we carried out ChIP studies to measure HA-tagged Ku70 association with an HO-induced DSB. In wild type cells we are unable to detect Ku enrichment near a DSB, indicating that any association this high-affinity DNA binding factor might have with the DSB must be extremely transient. In contrast, ChIP analysis detected a statistically significant Ku70 enrichment near the DSB in *mre11Δ* cells (Figure 4B), consistent with recent results obtained in *S. cerevisiae* [26], [59], [60]. Interestingly, we also observed a statistically significant increase in Ku enrichment in *ctp1Δ* cells, although not as high as in *mre11Δ* cells (Figure 4B). No enrichment of Ku at the DSB was observed in *exo1Δ* cells (Figure 4B), indicating that extended resection is not required to prevent Ku from associating with DNA ends. Combined with the genetic suppression of *mrnΔ* and *ctp1Δ* phenotypes by deleting Ku, these results suggest that MRN complex and Ctp1 are required to release the Ku heterodimer from DNA ends, and likely can prevent it from rebinding by creating resected DNA ends that are poor binding substrates for Ku. As seen for *ctp1Δ* mutants in fission yeast, Ku also accumulates at DSBs in *sae2Δ* mutants in *S. cerevisiae*, although the effect of a *sae2Δ* mutation is negligible in comparison to *mrxΔ* mutations [26], [59], [60].

These results suggested an epistatic relationship between Ctp1 and Mre11. Indeed, an IR survival assay confirmed that the *ctp1Δ mre11Δ* double mutant was no more sensitive than either single mutant (Figure S3A). As seen for the *ctp1Δ* and *mre11Δ* single mutants, deleting Ku made *ctp1Δ mre11Δ* cells more resistant to IR, and this suppression depended entirely on Exo1 (Figure S3A).

### Mre11 nuclease activity is not required for DSB resection in fission yeast

**Figure 5:**
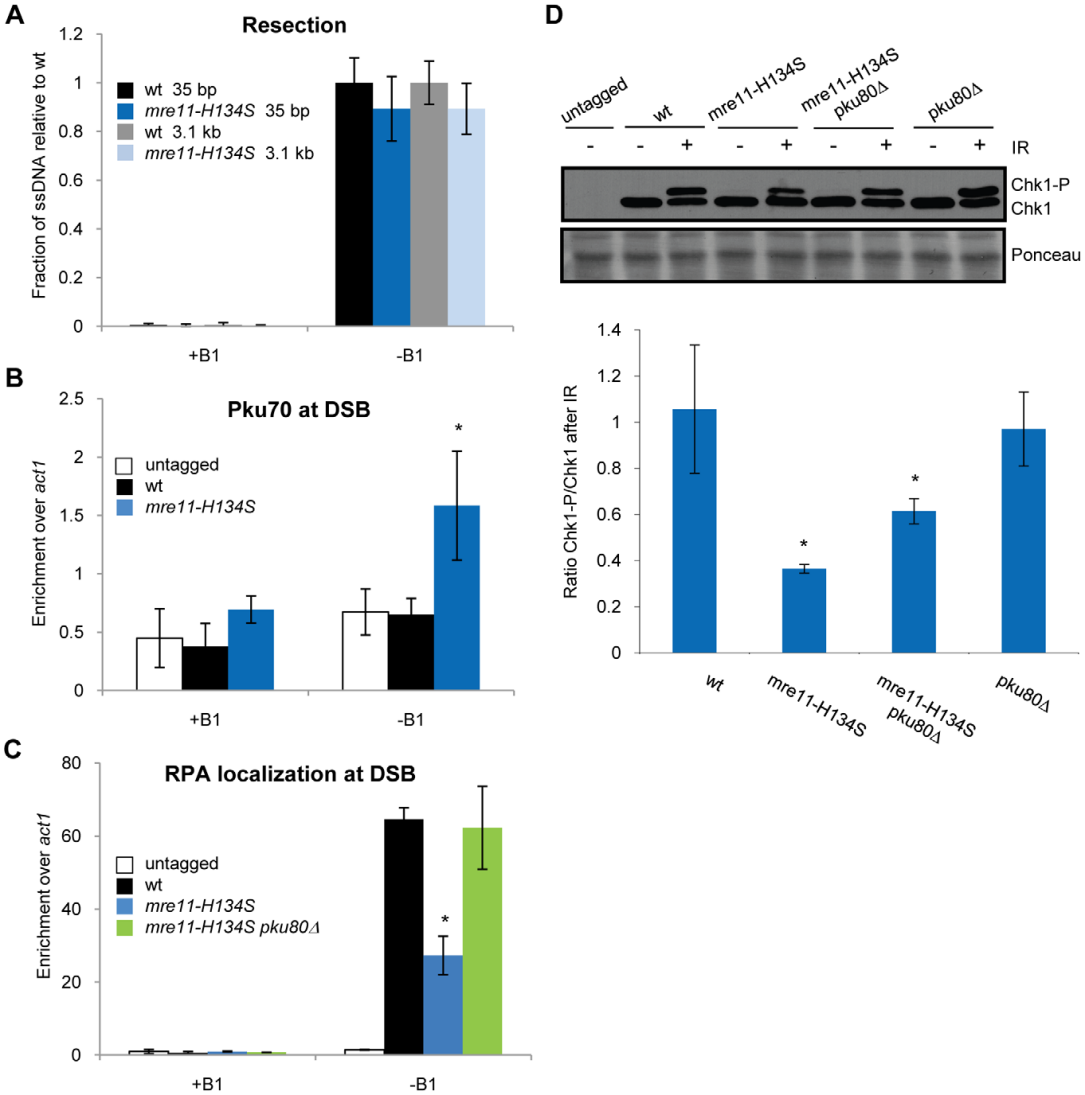
Functions of Mre11 nuclease activity in HR repair. Untagged and wild type values taken from previous figures, with the exception of D. +B1: HO endonuclease expression repressed. −B1: expression of HO endonuclease induced. A. Mre11 nuclease activity is not required for resection as ssDNA formation in *mre11-H134S* nuclease dead cells is not significantly decreased compared to wild type. B. Pku70-HA ChIP analysis shows increased Pku70 enrichment 0.2 kb from the HO endonuclease induced DSB in *mre11-H134S* cells compared to wild type. C. ChIP analysis of RPA (rad11-TAP) shows that RPA enrichment 0.2 kb from the HO endonuclease induced DSB in *mre11-H134S* cells is decreased compared to wild type. Deletion of Ku restores RPA localization to wild type levels. D. Chk1 undergoes strongly reduced activating phosphorylation (Chk1-P) 30 minutes after 90 Gy IR in *mre11-H134S* cells compared to wild type. Deletion of Ku increases Chk1 phosphorylation. Top: Western blot. Bottom: quantitation. Average and standard deviation (error bar) of three independent experiments are shown. Asterisk depicts statistically significant differences with wild type as determined by a two-tailed Student T-test, p-value≤0.05. In panel A: All values for the mutant strain are depicted relative to the average value of wild type. The wild type average was set to 1, with its standard deviation and values for the mutant adjusted by the same factor.

In *Schizo. pombe*, the *mre11-H134S* mutation that ablates Mre11 nuclease activity causes acute sensitivity to ionizing radiation, although not quite to the level of *mre11Δ* single mutants [46]. Mre11 nuclease activity is similarly critical for IR resistance in mammalian cells [49]. Mre11 nuclease activity appears to be much less important for IR resistance in *S. cerevisiae*, although defects are detected at high doses of IR [61]. Presented with these facts, we suspected that Mre11 nuclease activity might be important for resection in *Schizo. pombe*, and this might explain why the nuclease activity is required for radioresistance. Surprisingly, we found that the *mre11-H134S* nuclease-defective strain was not significantly defective in our resection assays (Figure 5A). Thus, Mre11 nuclease activity is not required for DSB resection in budding yeast or fission yeast. Some other DSB repair defect must therefore underlie the IR sensitivity of the *Schizo. pombe mre11-H134S* mutant.

### Ku is enriched at DNA ends in the absence of Mre11 endonuclease activity

When we measured the effect of deleting either Ctp1 or Mre11 on the enrichment of Ku at the DSB, we observed a higher enrichment of Ku in the *mre11Δ* than the *ctp1Δ* strain (Figure 4B, p-value≤0.05). As resection in these two strains is similarly affected, we hypothesized that Mre11 has an additional function that prevents Ku from binding or remaining attached to a DSB, compared to Ctp1. To test whether the additional function of Mre11 depends on its nuclease activity, we investigated Ku binding to the HO-induced DSB in *mre11-H134S* cells. We detected significant Ku enrichment, at a level similar to *ctp1Δ* cells (Figure 5B). These data are consistent with the suppression of *mre11-H134S* growth defects and DNA damage sensitive phenotypes by Ku deletion [46]. Thus, Ku accumulates at DSBs in *mre11-H134S* cells even though resection is proficient. These data appear to contrast with studies in *S. cerevisiae* that showed a negligible role for the Mre11 nuclease activity in regulating Ku accumulation at DNA ends [26], [59].

As discussed below, our data suggest that Mre11 nuclease activity and Ctp1 are both required to release Ku from DSBs. In agreement with this possibility, we found that a *ctp1Δ mre11-H134S* double mutant was no more sensitive to IR than either single mutant (Figure S3B).

### Reduced RPA localization at the DSB in *mre11-H134S* cells

Resection appears to be proficient in *mre11-H134S* cells (Figure 5A), and yet they are sensitive to IR and other DNA damaging agents. The IR sensitivity of *mre11-H134S* cells is suppressed by deleting Ku [46], and Ku accumulation at DSBs is elevated in *mre11-H134S* cells (Figure 5B). In an attempt to explain these results, we analyzed RPA localization at the HO break in *mre11-H134S* cells. Despite efficient resection, we found there was a significant defect in RPA enrichment in *mre11-H134S* cells (Figure 5C). Interestingly, just as seen for *ctp1Δ* and *mre11Δ* mutants, deletion of Ku increased RPA localization at the DSB in *mre11-H134S* cells. In fact, RPA enrichment at the DSB in *mre11-H134S pku80Δ* cells was not significantly different from wild type (Figure 5C). A similar relationship was observed when RPA enrichment was measured 2 kb from the DSB, although in this case deleting Ku in *mre11-H134S* cells did not fully restore the RPA signal to the wild type level (Figure S4A).

### Mre11 nuclease activity promotes Chk1 activation in irradiated cells

In view of the unexpected relationship between resection and RPA enrichment in *mre11-H134S* cells, we decided to measure a separate RPA-dependent response in these cells. The RPA-ssDNA filament recruits the heterodimeric Rad3-Rad26 checkpoint kinase that is orthologous to mammalian ATR-ATRIP complex [16]. Rad3 phosphorylates the checkpoint effector kinase Chk1 [62]. If RPA localization is reduced in *mre11-H134S* cells, Chk1 phosphorylation should be impaired as well. We used a well-established immunoblot assay to measure Rad3-dependent Chk1 phosphorylation in *mre11-H134S* and *mre11-H134S pku80Δ* cells [19], [57], [63]. Rather than creating an irreparable break with the HO endonuclease, we used IR to create DSBs that can be repaired by HR. Our analysis revealed a strong reduction in Chk1 phosphorylation in *mre11-H134S* cells compared to wild type 30 minutes after irradiation with 90 Gy (Figure 5D). This result is consistent with the reduction in RPA localization at the HO-induced DSB in these cells (Figure 5C). Elimination of Ku in *mre11-H134S* cells significantly increased Chk1 phosphorylation, although not to the level of wild type or *pku80Δ* cells (Figure 5D). The failure to fully restore Chk1 phosphorylation in *mre11-H134S pku80Δ* cells may reflect an inability to completely suppress defects in RPA localization, as suggested by ChIP assays performed at 2 kb from an HO-induced DSB (Figure S4A), or perhaps Mre11 nuclease activity has an additional role in promoting Chk1 activation at IR-induced DSBs.

### Ku-dependent enrichment of Mre11-H134S at DSBs

**Figure 6:**
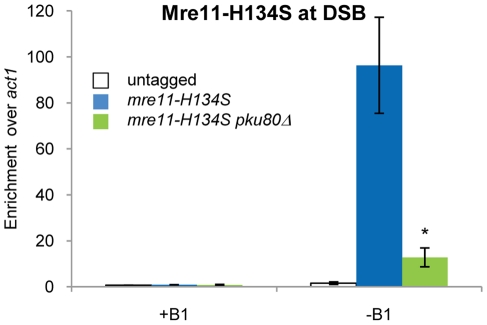
Ku stabilizes MRN binding on DNA ends in *mre11-H134S* cells. +B1: HO endonuclease expression repressed. −B1: expression of HO endonuclease induced. Enrichment of Mre11-H134S 0.2 kb from the HO endonuclease induced DSB in *mre11-H134S* cells can be reduced by deletion of Pku80. Average and standard deviation (error bar) of three independent experiments are shown. Asterisk depicts statistically significant differences with the *mre11-H134S* mutant as determined by a two-tailed Student T-test, p-value≤0.05.

If resection is proficient in *mre11-H134S* cells, why are RPA enrichment and checkpoint signaling reduced? We considered whether the presence of other proteins on the DNA might interfere with RPA localization. Ku was a candidate because structural studies showed that the Ku complex can translocate along duplex DNA to form a beads-on-a-string configuration [64], [65]. However, in *mre11-H134S* cells we could not detect Ku70 at 2 kb from the HO break (data not shown), even though RPA enrichment was reduced at this site (Figure S4A). Another candidate was the MRN complex. Our previous ChIP studies revealed that Mre11, Nbs1 and Ctp1 localize at the HO break in *mre11-H134S* mutant cells [46]. In fact, the ChIP signals appeared to be enhanced relative to wild type. Indeed, quantitative ChIP analysis revealed very strong enrichment of Mre11-H134S at the DSB (Figure 6). Crucially, this enrichment was largely ablated by deleting Ku (Figure 6). The same effect was observed at 2 kb from break (Figure S4B). Ku therefore appears to stabilize Mre11-H134S complex binding at DSBs, which likely interferes with efficient assembly of the RPA-ssDNA filament.

### Mre11 endonuclease activity and Ctp1 are required to overcome Ku interference with HR repair of a single site-specific broken replication fork

**Figure 7:**
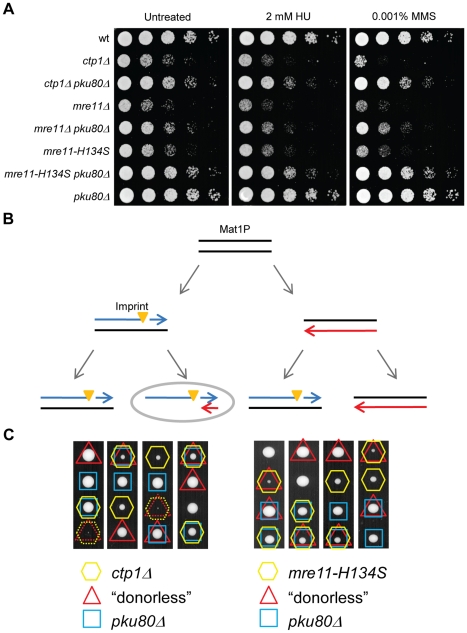
Mre11 nuclease, Ctp1, and Ku interplay in repair of a site–specific broken replication fork. A. In addition to desensitizing *ctp1Δ* and *mre11Δ* mutants to DSB inducing agents [19], [46], this dilution assay shows that deletion of Pku80 also protects these cells from damage induced by HU and MMS. B. Mating type pedigree is shown. The first round of cell division creates one daughter with a single strand break, called the imprint (orange triangle). Further cell division of this daughter will result in a single one-ended DSB. Newly synthesized DNA: blue and red arrows. Cell with DSB: grey oval. C. Tetrad dissection of a mating between *ctp1Δ pku80Δ* (left panel) or *mre11-H134S pku80Δ* (right panel) and *mat1-P(2,3Δ)* or “donorless” strains. Replication forks collapse near the mating type locus in donorless strains. The collapsed replication fork is restored by a homologous recombination-dependent mechanism requiring Ctp1, and to a lesser extent, the nuclease activity of Mre11. This requirement is overcome by deletion of Ku.

Our data suggest that Mre11 endonuclease activity and Ctp1 are required to effectively release Ku and MRN complex from DSBs. As genetic deletion of Ku substantially alleviates the poor growth and genotoxin sensitivity of *ctp1Δ* and *mre11-H134S* mutants, not only to IR and CPT, but also to compounds such as hydroxyurea (HU) and methyl methanesulfonate (MMS) that indirectly cause DSBs (Figure 7A), our data suggest that Ku release is a critical function of Mre11 endonuclease activity and Ctp1. But why is the release of Ku crucial if NHEJ is an alternative mechanism for the repair of DSBs? This question is particularly relevant to mammalian cells in which NHEJ appears to be responsible for the bulk of DSB repair. An answer to this question may lie with the repair of broken replication forks, which are likely the most abundant form of spontaneous DSBs. Unlike the two-ended DSBs made by ionizing radiation or by the HO endonuclease, broken replication forks create one-ended DSBs that cannot be repaired by NHEJ [66]. Mre11 and Ctp1 may therefore function to protect cells from the detrimental effects of Ku DNA end-binding on the repair of one-ended DSBs. To test this hypothesis, we made use of the mating type locus in which a stalled replication fork creates a single one-ended rather than two-ended DSB (Figure 7B) [67]–[69]. Using an altered version of the mating type locus, which lacks homologous donor sequences for repair by gene conversion (*mat1-P(2,3Δ)* or “donorless” strain), we previously determined that Rad50, one of the members of the MRN complex, is required for survival of this strain [70]. Similarly, we found that the *ctp1Δ mat1-P(2,3Δ)* cells grew extremely poorly (Figure 7C). The defect was similar but slightly weaker in *mre11-H134S mat1-P(2,3Δ)* cells (Figure 7C). Importantly, deletion of Ku substantially enhanced growth in these genetic backgrounds (Figure 7C). From these results we conclude that Ctp1 and Mre11 nuclease activity are required to overcome the inhibitory effects of Ku on HR repair of one-ended DSBs formed by replication fork collapse in S-phase.

## Discussion

In this study we have investigated the roles of Mre11 endonuclease activity and Ctp1 in DNA end resection and DSB repair. Our goal was to understand why these activities are crucial for DSB repair in fission yeast, and why the requirement for their critical functions can be bypassed by eliminating the Ku complex. By establishing a resection assay that measures ssDNA formation in *Schizo. pombe*, we found that resection frequently fails to initiate at irreparable HO-induced DSBs in *ctp1Δ* mutants (Figure 2A). This defect likely explains much of the extreme IR sensitivity of these cells, as resection is essential for HR repair of DSBs, and HR is essential for IR survival. The same defect was observed in an *mre11Δ* mutant (Figure 2A). Mre11 is required to recruit Ctp1 to DSBs [19], which by itself could explain the IR sensitivity of *mre11Δ* cells, although Mre11 also contributes other functions that are critical for DSB repair. One of these activities is Mre11 nuclease function, as *mre11-H134S* mutants are very sensitive to IR, and yet there is no defect in Ctp1 recruitment to DSBs in these cells [46], nor is there a measurable defect in resection (Figure 5A). Rather, our studies show that *ctp1Δ*, *mre11Δ* and *mre11-H134S* cells all accumulate the Ku heterodimer at DNA ends (Figure 4 and Figure 5B). From these data we conclude that the activities of the MRN complex and Ctp1 are required to release the Ku heterodimer from DNA ends to overcome the inhibitory effect of Ku on HR repair of DSBs. By combining the data from the resection assay with the results of our RPA ChIP experiments, we are able to study the interplay between the proteins involved in resection and the localization of RPA to ssDNA during the repair of DSBs *in vivo*. Intriguingly, despite the seemingly unaffected resection status of *mre11-H134S* mutant cells, we find a defect in the localization of RPA that can be corrected by deleting Ku (Figure 5C). It appears that Ku stabilizes the binding of MRN to a DSB in *mre11-H134S* cells, resulting in enhanced Mre11-H134S occupancy on the ssDNA, which interferes with RPA assembly on ssDNA. As a consequence, *mre11-H134S* cells exhibit reduced checkpoint activation after irradiation (Figure 5D) and decreased resistance to DSB-inducing agents. Furthermore, our data indicate that the decreased ability to release Ku and MRN from DNA ends in *mre11-H134S* cells, and the resulting reduction in RPA localization, causes substantial sensitivity to a single one-ended DSB formed by replication fork collapse (Figure 7). When these defects are combined with reduced resection, as seen in *ctp1Δ* cells, the result is nearly 100% lethality in cells experiencing a single collapsed replication fork (Figure 7).

### Ctp1, Mre11, and Exo1 in resection in fission yeast

Ctp1 and Mre11 are critical to efficiently initiate resection of DSBs (Figure 2A). Extended resection measured at 3.1 kb from the DSB is mainly achieved through the activity of Exo1 (Figure 2D). Only in the absence of Exo1 do we uncover a modest contribution of Rqh1 (Figure 2D). These data are generally consistent with studies in budding yeast using Southern blot-based assay systems. The role of Sae2 was initially detected in an *exo1Δ sgs1Δ* background, indicating that the initial ∼100 bp cleavage is made by Mre11 complex and Sae2 [27], [28]. Ensuing resection up to ∼5 kb from the DSB is slowed in the absence of Exo1 or Sgs1, although it is necessary to eliminate both Exo1 and Sgs1 to effectively block long-range resection [27], [28]. However, resection in the absence of Sae2 is only mildly affected, suggesting that Exo1 or Sgs1-dependent activities can effectively initiate resection, and this likely explains why *sae2Δ* mutants are only mildly radiosensitive compared to MRX mutants [6], [26], [39], [41].

In an effort to completely eliminate resection, we tried to generate *ctp1Δ exo1Δ rqh1Δ* triple mutants. We found that the triple mutant is unviable (Figure S2). We were also unable to analyze resection in *ctp1Δ rqh1Δ* mutants, as the break induction kinetics in this extremely sick strain is slower than that of all other strains, making comparisons between strains impossible. Similar synthetic growth defects and lethality have been observed in budding yeast [27].

### The DNA end-binding capacity of Ku interferes with initiation of resection by Exo1

Deleting Ku substantially rescues the genotoxin sensitivity of *mre11Δ*, *mre11-H134S*, *rad50Δ* and *ctp1Δ* mutants [19], [46], [58], [71]. This rescue requires Exo1, indicating that Ku prevents Exo1 from substituting for the MRN complex and Ctp1. Indeed, deleting Ku in *ctp1Δ* cells enhances Exo1-dependent initiation of resection (Figure 2F). Similar results were observed in budding yeast, where deleting Ku suppressed the initial resection delay in *sae2Δ* cells and improved resection in an Exo1-dependent manner in a *rad50Δ* mutant [26], [72]. Additionally, Exo1 was shown to be involved in both the initiation and extension of resection at a *VMA1* derived homing endonuclease (VDE)-induced DSB during meiosis in budding yeast [73].

We observed no defect in ssDNA formation at 35 bp or 3.1 kb from the DSB in the *mre11-H134S* mutant, consistent with observations in *S. cerevisiae* [50]. However, if Mre11 nuclease activity is not required for resection, then why is Exo1 required for robust growth and radioresistance in *mre11-H134S* cells, both in the presence or absence of Pku80? These data suggest that Exo1 might be important for resection in the absence of Mre11 nuclease activity. In budding yeast cells lacking Mre11 nuclease activity, Exo1 is not required for resection, but resection initiation is substantially impaired in the absence of Sgs1 and completely abrogated in when both Sgs1 and Exo1 are eliminated [26], [72]. These observations suggest that resection activities that are normally involved in extended resection can be essential for resection initiation in the absence of Mre11 nuclease activity.

### Ku enriches at DNA ends in the absence of Mre11 nuclease activity and Ctp1, which results in stabilized MRN binding and coincides with a decrease in RPA assembly on ssDNA

Ku is enriched at DNA ends in the absence of Mre11 nuclease activity or Ctp1 (Figure 4B and Figure 5B). While Ku's affinity for blunt DSBs can explain this effect in resection-defective *ctp1Δ* cells, the same explanation cannot easily account for Ku enrichment in resection-proficient *mre11-H134S* cells (Figure 2A and Figure 5A). The requirement for both Mre11 nuclease activity and Ctp1 to release Ku may have some analogy to the release of Rec12^Spo11^ that is covalently bound to DSBs in meiosis, although removal of Rec12^Spo11^ is required for efficient resection [44]. Mre11 nuclease activity and Ctp1 are required to release Rec12 bound to oligonucleotides that range roughly from 13 to 29 nucleotides [42], [43]. Our resection assay shows that while Mre11-H134S and Ku still bind DNA in *mre11-H134S* cells, resection at 35 bp is comparable to wild type. As the Ku heterodimer can bind to dsDNA regions as short as 25 bp [74], one possibility is that there is a region of dsDNA adjacent to the HO break, which is smaller than 35 bp, that retains Ku. We are currently investigating this idea.

The necessity to release Ku from the DNA to allow HR repair can explain the critical requirement for Ctp1 and Mre11 nuclease activity in HR in fission yeast [19], [46]. In contrast, removing Sae2 or the Mre11 nuclease function in budding yeast has little effect on resection or the release of Ku [6], [26]–[28], [59], which explains why they are largely dispensable for HR in budding yeast [39], [41], [47], [48]. We observe a stronger enrichment of Ku at the DNA end in the absence of Mre11 than in the absence of either Ctp1 or Mre11 nuclease activity alone (Figure 4B). This increased enrichment of Ku in *mre11Δ* cells can be explained either by decreased competition between MRN and Ku for DNA ends, or by the combined lack of Mre11 nuclease activity and Ctp1, as Ctp1 depends on Mre11 for recruitment to DSBs [19].

RPA localization to naked ssDNA is near diffusion limited when assayed *in vitro* with purified components [75], but our data suggest that *in vivo*, localization of RPA to ssDNA can be influenced by other factors. Interestingly, the reduced localization of RPA observed in *mre11-H134S* cells is reflected in human and murine cells expressing nuclease dead Mre11, as RPA foci formation following IR was strongly reduced in these mutant cells compared to wild type [49], [76]. This suggests that the function of Mre11 nuclease activity to enable RPA localization is conserved in these species.

Reduced RPA localization at a DSB in *Schizo. pombe mre11-H134S* cells depends on Ku (Figure 5C). As we have shown, both Ku and Mre11-H134S are enriched at the DNA end in *mre11-H134S* cells (Figure 5B and Figure 6). Other proteins occupying the ssDNA, thereby preventing RPA from binding, might therefore explain the reduced RPA localization observed in *mre11-H134S* cells. One candidate is the MRN complex itself, as the Mre11-H134S ChIP signal at a DSB is high in an *mre11-H134S* background, but is reduced when Ku is deleted (Figure 6). It is of interest to note that this observation strongly suggests that Ku and MRN can bind to the same DNA end. Our data suggest that Ku either traps MRN to the DNA, or that it inhibits the action of an unidentified nuclease that enables the release of MRN from the DNA. A possible candidate is Exo1, as rescue of the poor growth and genotoxin sensitivity of *mre11-H134S* mutants by deletion of Pku80 is dependent on Exo1 [46]. However, the most likely candidate is Ctp1, as deletion of Ctp1 increases and prolongs MRN binding to DNA at a HO-induced DSB in wild type cells [57]. Similar effects on MRX foci formation were observed in the absence of Sae2 at a HO-induced DSB in budding yeast [77].

RPA localization in *ctp1Δ pku80Δ* and *mre11Δ pku80Δ* mutant cells is significantly lower than that observed in the *pku80Δ* mutant (Figure 3, data not shown for *mre11Δ pku80Δ*), while resection in *ctp1Δ pku80Δ*, *mre11Δ pku80Δ* and *pku80Δ* mutants are indistinguishable (Figure 2E). This suggests that other proteins facilitate RPA localization at ssDNA *in vivo*. One possibility is Mre11, as several studies in mammalian cells have shown an interaction between Mre11 and RPA [78]–[80]. We are currently investigating this interaction in *Schizo. pombe*.

### Roles of Mre11 nuclease activity and Ctp1 in HR repair of DSBs

**Figure 8:**
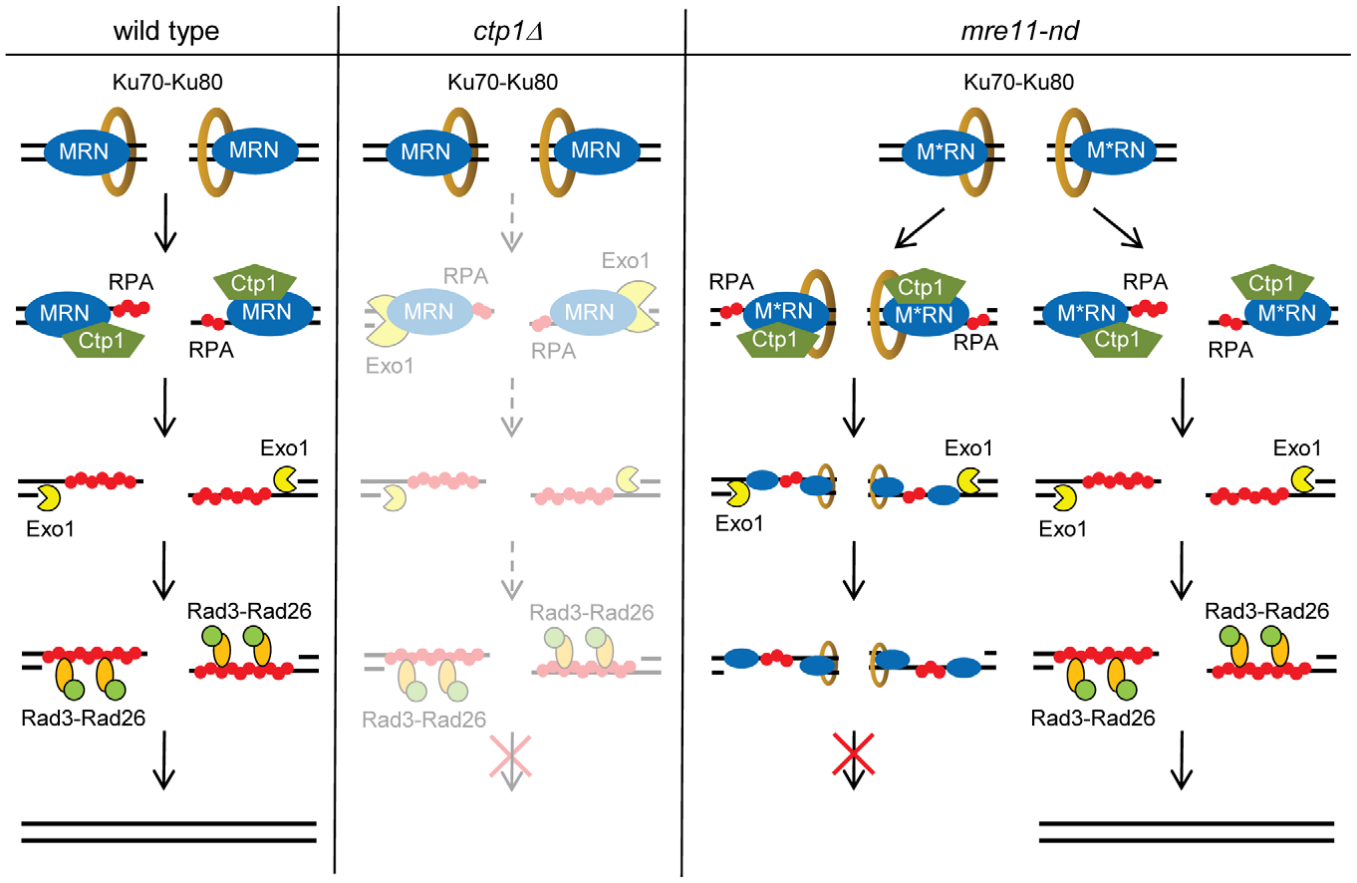
Model for resection in *Schizo. pombe*. Wild type: Ku and MRN recognize the DSB, and MRN recruits Ctp1. Ctp1 initiates resection. The action of the Mre11 nuclease and Ctp1 releases Ku from the DNA end, after which MRN is released from the DNA. Resection can be extended by Exo1. Localization of RPA to ssDNA recruits Rad3-Rad26 and activates the checkpoint pathway by phosphorylating Chk1. The break is repaired. *Ctp1Δ*: MRN recognizes the DSB, but absence of Ctp1 inhibits release of Ku and resection initiation. Inefficient Ku release by Mre11 nuclease allows Exo1 to substitute for Ctp1 in the initiation of resection at some DNA ends. Exo1 also extends resection. This step is inefficient and results in strongly decreased RPA binding to the ssDNA and prevents HR repair. *Mre11-ND*: M*RN recognizes the DSB, and recruits Ctp1. The lack of the nuclease function of Mre11 inhibits Ku release. Ctp1 can initiate resection for several hundred base pairs, allowing Exo1 to extent resection despite the presence of Ku on the DNA end. The inability to release Ku and M*RN from the DNA end interferes with RPA localization at the ssDNA and prevents repair. Ctp1 is however able to release Ku from some DNA ends. Resection and efficient RPA binding to the ssDNA overhangs allows repair of the DSB.

Our findings provide evidence for some of the underlying causes that result in the observed slow growth and DNA damage sensitivity phenotypes of *mre11-H134S* and *ctp1Δ* mutants in *Schizo. pombe*. DSBs can be recognized by MRN alone, Ku alone or both MRN and Ku. DSBs recognized by MRN alone do not provide difficulties for repair by homologous recombination. On the other hand, when the DSBs are recognized by Ku alone, the breaks may be repaired by NHEJ, or alternatively, repair is prevented by inhibiting the initiation of resection. Our observations that both Ku and MRN can be enriched at DNA ends in *mre11-H134S* and *ctp1Δ* cells (Figure 4B, Figure 5B, Figure 6, and Figure S4B and [57]), and that stabilization of MRN binding depends on the enrichment of Ku (Figure 6 and Figure S4B) suggest that there are a significant number of DNA ends that are recognized by both protein complexes at the same time. Based on our results, we propose that MRN recruits Ctp1 to these DSBs, after which the combined activities of Ctp1 and Mre11 nuclease ensure the release of Ku from the DNA end. The same activities are required for the release of the MRN complex from the DSB. Resection can be initiated by Ctp1 and extended by Exo1, which can function independently of Ctp1. Extension of resection by Exo1 is however likely not absolutely required, as *exo1Δ* cells are not sensitive to DSB inducing agents [19]. RPA bound to the generated ssDNA overhangs will recruit Rad3-Rad26, resulting in activation the checkpoint pathway by phosphorylation of Chk1 (see Figure 8).

In the absence of Ctp1 however, Mre11 nuclease activity is not efficient in releasing Ku and MRN from DNA ends and resection is often not initiated. Release of Ku from some DNA ends may allow Exo1 to substitute for Ctp1 in the initiation of resection. The strongly decreased RPA binding observed in *ctp1Δ* mutant cells suggests that this step is highly inefficient (see Figure 8). This is supported by our observation that a single collapsed replication fork is lethal in a *ctp1Δ* mutant (Figure 7), which likely is the result of a combination of reduced resection and defects in release of Ku and the MRN complex.

On the other hand, when Mre11 nuclease activity is absent, Ctp1 can still be recruited [46] and initiate resection at all DNA ends. However, Ctp1 cannot release Ku from all DNA ends in the absence of Mre11 nuclease activity, which will create a situation where some DNA ends are bound by Ku and MRN, while other DNA ends are free from protein complexes (exemplified in Figure 8). We envision that Ctp1 can initiate resection up to several hundred base pairs, allowing Exo1 to extend resection on the 5′ recessed DNA ends despite the binding of Ku to the DNA end. This explains why resection is unaffected in *mre11-H134S* cells. The defective release of the MRN complex in *mre11-H134S* cells in the presence of Ku results in reduced RPA localization, less efficient checkpoint activation, and inefficient repair of the DSB (see Figure 8). On the other hand, RPA is able to bind to the free ssDNA overhangs resulting in repair of the DSB by homologous recombination (see Figure 8), which provides an explanation for the observation that a single collapsed replication fork decreases cell viability, but is not lethal in *mre11-H134S* cells (Figure 7).

The model in Figure 8 does not propose an explanation for the observed small decrease in resection initiation in the absence of Ku. This observation may reflect a MRN-recruiting function of Ku. However, the physiological significance of this defect is unclear, as *pku80Δ* mutants appear to be fully proficient at RPA binding and HR repair of DSBs (Figure 3 and [5]). An interaction between Mre11 and Pku80 has been detected in *S. cerevisiae* [81] and we are currently investigating this interaction in *Schizo. pombe*.

Our data clearly show that DNA end resection is insufficient to initiate HR repair of DSBs — in addition to generation of ssDNA overhangs through resection initiated by Ctp1 and the MRN complex, Ctp1 and Mre11 nuclease activity must release MRN and Ku complexes to allow efficient RPA localization at resected DSBs. Therefore, our data indicate that while RPA localization does not always reflect the resection status of DSBs, it always reflects the ability to repair: inefficient RPA localization despite proficient resection leads to inefficient repair and therefore sensitivity to DSB inducing agents. We hypothesize that the relationships between MRN, Ctp1 and Ku are conserved in mammals, and this may explain why Mre11 endonuclease activity and CtIP are critical for cellular viability in mice [49], [82].

Interestingly, our data describing the functions of Mre11, Ctp1 and Exo1 in resection of DSBs in mitotic cells are reminiscent of a study showing the requirements of the same proteins for repair of an *I-Sce*-induced DSB during meiosis [83]. This study showed that Ctp1 is required for repair in the presence of the MRN complex, while Exo1 is required when MRN is absent [83]. Our resection data provide a possible explanation for this observation: in the presence of MRN, Ctp1 is recruited to DSBs and initiates resection. In the absence of MRN, Ctp1 is no longer recruited and Exo1 will initiate resection. There are however also clear differences: in meiotic cells MRN appears to inhibit Exo1, while in mitotic cells it is Ku that inhibits Exo1 activity. The lack of an inhibitory effect of Ku on repair of DSBs in meiotic cells would bypass the need for Mre11 nuclease-dependent release of Ku from DNA ends. Indeed, Mre11 nuclease activity is not required for repair of an *I-Sce*-induced DSB during meiosis in fission yeast [83], while it is required for repair of IR-induced DSBs in mitotic cells [46]. Curiously, in budding yeast Mre11 nuclease activity is required for resection of a VDE-induced DSB during meiosis [73]. The reasons for the different requirements for Mre11 nuclease activity between fission and budding yeast, and mitosis and meiosis remain elusive.

### Remaining questions

While our studies provide important insights into the interplay of NHEJ and HR factors at DSBs, and point out some key similarities and differences between *Schizo. pombe* and *S. cerevisiae*, they also raise new questions. On the face of it, the relationship between Ctp1, Mre11 and Ku is counter-intuitive, as NHEJ would be expected to be critical for survival of DSBs in these HR-defective strains. However, we and others found that deleting two other essential NHEJ factors, Xlf1/Cernunnos or Ligase IV, does not suppress *mre11* or *ctp1Δ* mutants [58], and thus inactivating NHEJ is insufficient for suppression. Instead, the high-affinity binding of Ku to DNA is sufficient to inhibit HR in these mutants, even if later steps of NHEJ are blocked. These data point to a competition between the Ku heterodimer and the MRN-Ctp1 unit to determine whether NHEJ or HR repairs DSBs. Ku might bind DNA ends first and then is subsequently released from the DNA through the DNA end processing activities provided by the MRN complex and Ctp1. Ku has a very high affinity for DNA ends, and yet clearly NHEJ cannot efficiently repair DSBs in *mre11-H134S* or *ctp1Δ* mutants. Why this is so remains a mystery. It is possible that the MRN complex promotes NHEJ repair of chromosomal DSBs in fission yeast, as the MRX complex does in budding yeast [84], [85], but the failure to release the MRN complex in *mre11-H134S* and *ctp1Δ* mutants likely interferes with the completion of NHEJ. The requirement for the active release of Ku, a protein complex showing very high affinity for and slow dissociation from DNA ends [60], [86], becomes more evident when NHEJ cannot be considered as an alternative pathway for repair, such as is the case with one-ended DSBs that can arise from stalled or broken replication forks.

## Materials and Methods

### General methods

All strains used in this study harbor a single HO cleavage site at the *arg3* locus and carry a gene coding for a thiamine (B1) -repressible HO endonuclease as previously described [53]. Strains are listed in Table S1. Cells were grown in YES unless stated otherwise. Plate survival assays were performed using 5× serial dilutions. For IR survival cells were exposed to 90 Gy IR using a ^137^Cs source. Plates were analyzed after an incubation period of three days at 30°C. For HO endonuclease induction pre-cultures were grown up to 28 hours, washed three times and subsequently cultured in medium without B1. Samples were taken hourly, starting after 12 hours. Immunoblotting was performed as previously described [19]. Immunoblots were quantified with ImageJ software (http://rsb.info.nih.gov/ij/). Error bars represent the standard deviation of three independent experiments.

### DNA isolation and quantitative real-time PCR

10 ml of cells from a saturated culture were resuspended in 200 µl of genomic DNA extraction buffer (2% Triton X100, 1% SDS, 100 mM NaCl, 10 mM Tris-HCl pH 8.0, 1 mM EDTA). After addition of 200 µl Phenol∶Chloroform∶Isoamylalcohol (Sigma Life Sciences) and glass beads, cells were lysed by vortexing 5 minutes at 4°C. DNA was then prepared by standard phenol/chloroform extraction. RNA was removed by addition of RNase A (1 hour at 37°C). 35 ng of DNA was used for each quantitative real-time PCR reaction, and all reactions were performed in triplicate. We used the iQ SYBR Green Supermix with the Chromo4 Real-Time PCR Detection System (Bio-Rad Laboratories). The following program was used: 95°C for 3 min, 37× (95°C 15 s, 60°C 15 s, 72°C 30 s). Primer sequences are listed in Table S2. To determine the efficiency of the HO break induction, we performed standard curves with primer pairs 1 and 2. We then calculated the fraction of uncut DNA by measuring the total amount of DNA (primer pair 1) and the amount of uncut DNA (primer pair 2)(see Figure S1A). To calculate the percentage of ssDNA at each time point we used the method described by [51] with the following exception: digestion of the DNA was performed with *ApoI*. Resection was measured at two distances from the break based on the location of the *ApoI* restriction site: 35 bp (primer pair 3) and 3.1 kb (primer pair 4) respectively (see Figure S1B). To determine the overall rate of resection in a wild type strain, we measured resection at three addition distances from the break: 270 bp (primer pair 5), 5.4 kb (primer pair 6) and 9.4 kb (primer pair 7). The HO endonuclease is induced at different times after removal of B1 depending on the strain used. To directly compare the resection between the different strains, we overlay the graphs showing the percentage of uncut DNA (see Figure S5). Kinetics of resection can be found in Figure S6. Table S3 shows the range of the percentage of uncut DNA for each strain at the “−B1” point as used in Figure 2.

### Chromatin immunoprecipitation assays

ChIP assays were performed as previously described [53] with the following modifications: IgG Sepharose 6 Fast-Flow beads (GE Healthcare) were used to retrieve TAP-tagged Rad11 and mouse anti-HA antibody (Roche Applied Science) conjugated to anti-mouse magnetic beads (Dynal/Invitrogen) were used to pull down Pku70-HA, while mouse anti-myc antibody (Roche Applied Science) conjugated to anti-mouse magnetic beads (Dynal/Invitrogen) were used to pull down Mre11-H134S-myc. Primers used to measure protein enrichment at 0.2 kb from the break are listed in [53]. Purelink PCR Purification Kit (Invitrogen) was used to recover DNA. In all ChIP assays cells were checked for HO break induction by measuring the percentage of uncut DNA as described above. The induced timepoint shown in all the graphs depicting ChIP data is the timepoint at which we observe maximal enrichment of the protein of interest in the strain with the highest enrichment: for RPA: wild type, for Mre11-H134S: *mre11-H134S* mutant and for Ku: *mre11Δ* mutant.

## Supporting Information

Figure S1 qPCR strategy to measure resection in *Schizo. pombe*. A. Schematic overview for the detection of uncut DNA. The fraction of uncut DNA is calculated by measuring the total amount of DNA (primer pair 1) and the amount of uncut DNA (primer pair 2) to determine the efficiency of the break induction. B. Resection generates ssDNA, which is protected from digestion by restriction enzymes. To calculate the percentage of ssDNA at each time point, we isolate the DNA and either digest with *ApoI* or mock-treat the sample. Resection was measured at two distances from the break based on the location of the *ApoI* restriction site: 35 bp (primer pair 3) and 3.1 kb (primer pair 4) respectively.
(TIF)

Figure S2 Triple mutants of *exo1Δ rqh1Δ* and *ctp1Δ*, *mre11Δ* or *mre11-H134S* are synthetic lethal. Tetrad dissections of mating between *exo1Δ rqh1Δ* and *exo1Δ ctp1Δ*, *exo1Δ mre11Δ* or *exo1Δ mre11-H134S* mutants respectively reveal that triple mutants are unviable.
(TIF)

Figure S3 Epistasis analysis of Ctp1 and Mre11. A. Dilution assay depicting that deletion of Ctp1 is epistatic with deletion of Mre11 with regard to the slow growth and IR sensitivity. Deletion of Pku80 increases the fitness and radioresistance of *mre11Δ ctp1Δ* double mutants as it does in either single mutant. The rescue by Pku80 deletion requires Exo1. B. Dilution assay depicting that removal of Mre11 nuclease activity is epistatic with deletion of Ctp1 with regard to slow growth and IR sensitivity.
(TIF)

Figure S4 Ku-dependent stabilization of MRN binding decreases RPA localization 2 kb from the DNA end. A. ChIP analysis of RPA (rad11-TAP) shows that RPA enrichment 2 kb from the HO endonuclease induced DSB in *mre11-H134S* cells is decreased compared to wild type. Deletion of Ku significantly, but partially restores RPA localization compared to wild type. B. Enrichment of Mre11-H134S 2 kb from the HO endonuclease induced DSB in *mre11-H134S* cells can be reduced by deletion of Pku80. Average and standard deviation (error bar) of three independent experiments are shown. Asterisk depicts statistically significant differences with wild type (A) or the *mre11-H134S* mutant (B) as determined by a two-tailed Student T-test, p-value≤0.05.
(TIF)

Figure S5 Overlay of break inductions of all strains used. The HO endonuclease is induced at different times after removal of B1 depending on the strain used, sicker strains can take up to 5 hours longer to induce than healthier strains. To directly compare the resection between the different strains, we shift the time course of each strain, to form the most perfect overlay of HO induction as measured by the percentage of uncut DNA. “+B1” represents the repressed condition (HO endonuclease off), time point 1 is the first time point at which the percentage of uncut DNA is lower than 95%. Induction of the HO endonuclease is shown for all strains.
(TIF)

Figure S6 Kinetics study for resection. “+B1” shows the percentage of ssDNA immediately before removal of thiamine. Resection is followed for four hours (until the maximum of cut DNA is reached) from the time point that the percentage of ssDNA in wild type reaches 10%. Averages and standard deviation (error bar) of at least three independent experiments are shown.
(TIF)

Table S1 *Schizo. pombe* strains used in this study.
(DOC)

Table S2 Primers used in this study.
(DOC)

Table S3 Range of the percentage of uncut DNA for each strain at “−B1” as used in Figure 2.
(DOC)
