# Supplementary figures and images for: Release of Ku and MRN from DNA Ends by Mre11 Nuclease Activity and Ctp1 Is Required for Homologous Recombination Repair of Double-Strand Breaks
Source: PLoS Genet. 2011 Sep 8;7(9):e1002271. doi: 10.1371/journal.pgen.1002271 (PMC3169521; doi:10.1371/journal.pgen.1002271)

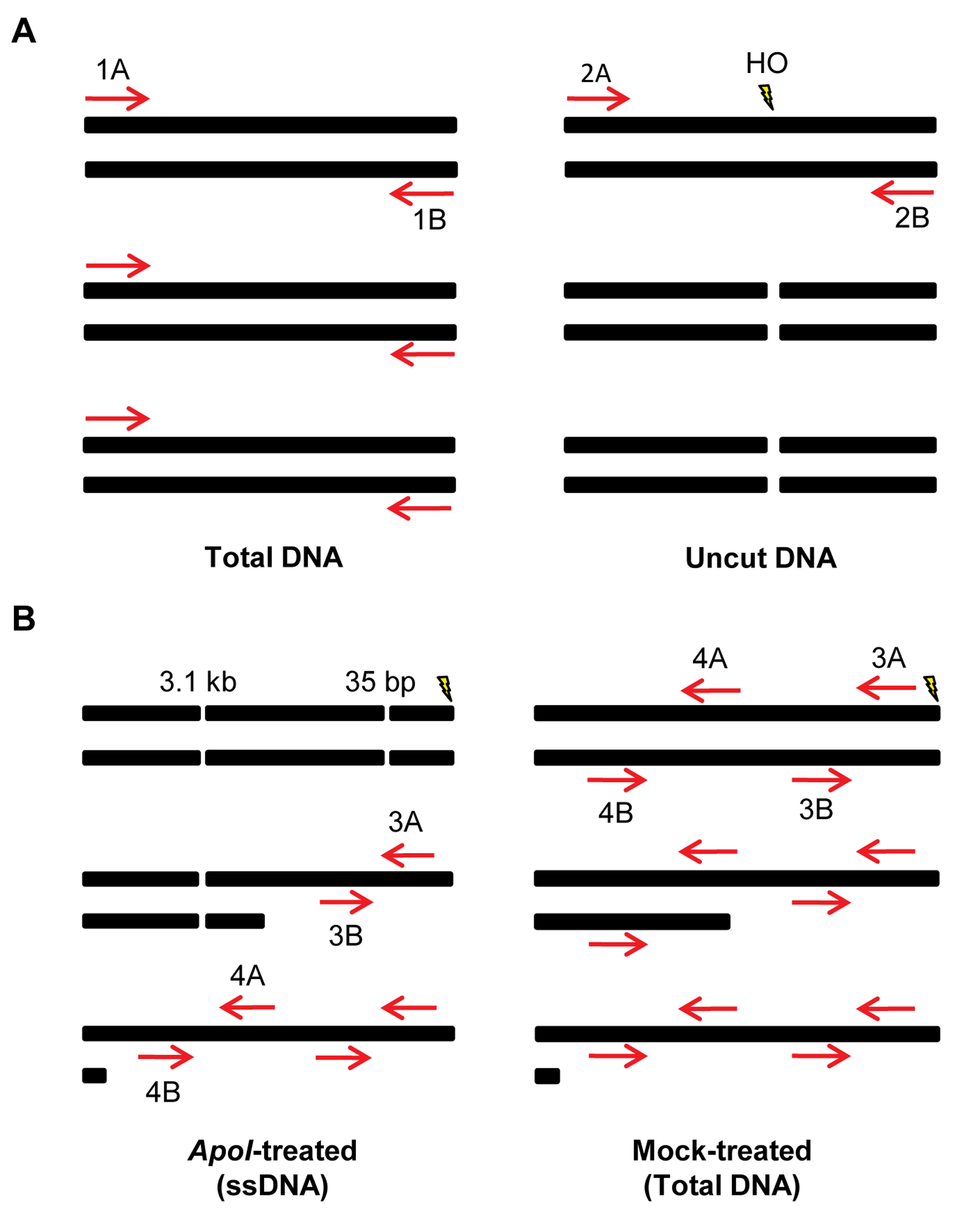

Supplement: Figure S1 — qPCR strategy to measure resection in Schizo. pombe. A. Schematic overview for the detection of uncut DNA. The fraction of uncut DNA is calculated by measuring the total amount of DNA (primer pair 1) and the amount of uncut DNA (primer pair 2) to determine the efficiency of the break induction. B. Resection generates ssDNA, which is protected from digestion by restriction enzymes. To calculate the percentage of ssDNA at each time point, we isolate the DNA and either digest with ApoI or mock-treat the sample. Resection was measured at two distances from the break based on the location of the ApoI restriction site: 35 bp (primer pair 3) and 3.1 kb (primer pair 4) respectively. (TIF) [file pgen.1002271.s001.tif]

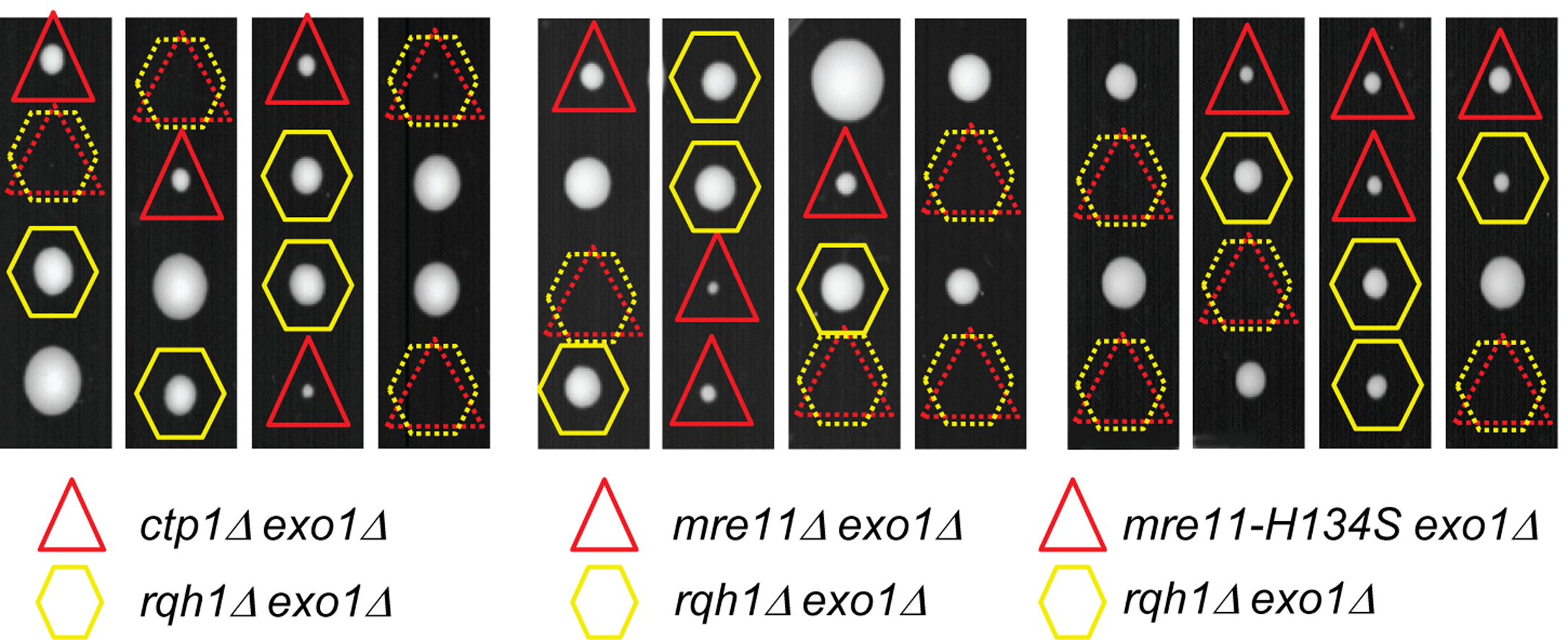

Supplement: Figure S2 — Triple mutants of exo1Δ rqh1Δ and ctp1Δ, mre11Δ or mre11-H134S are synthetic lethal. Tetrad dissections of mating between exo1Δ rqh1Δ and exo1Δ ctp1Δ, exo1Δ mre11Δ or exo1Δ mre11-H134S mutants respectively reveal that triple mutants are unviable. (TIF) [file pgen.1002271.s002.tif]

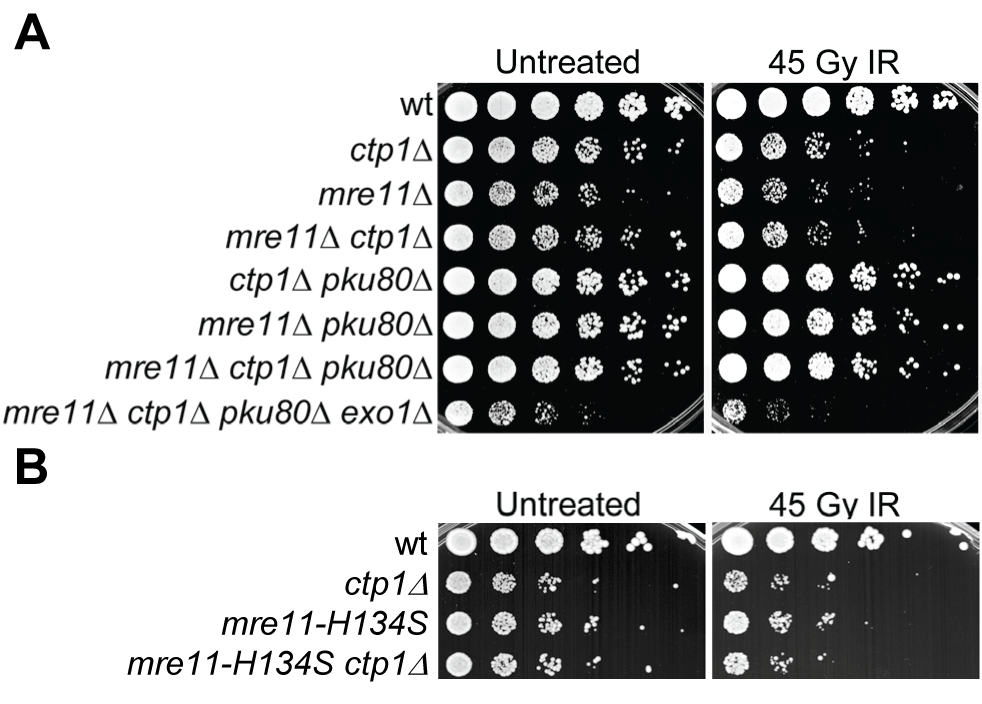

Supplement: Figure S3 — Epistasis analysis of Ctp1 and Mre11. A. Dilution assay depicting that deletion of Ctp1 is epistatic with deletion of Mre11 with regard to the slow growth and IR sensitivity. Deletion of Pku80 increases the fitness and radioresistance of mre11Δ ctp1Δ double mutants as it does in either single mutant. The rescue by Pku80 deletion requires Exo1. B. Dilution assay depicting that removal of Mre11 nuclease activity is epistatic with deletion of Ctp1 with regard to slow growth and IR sensitivity. (TIF) [file pgen.1002271.s003.tif]

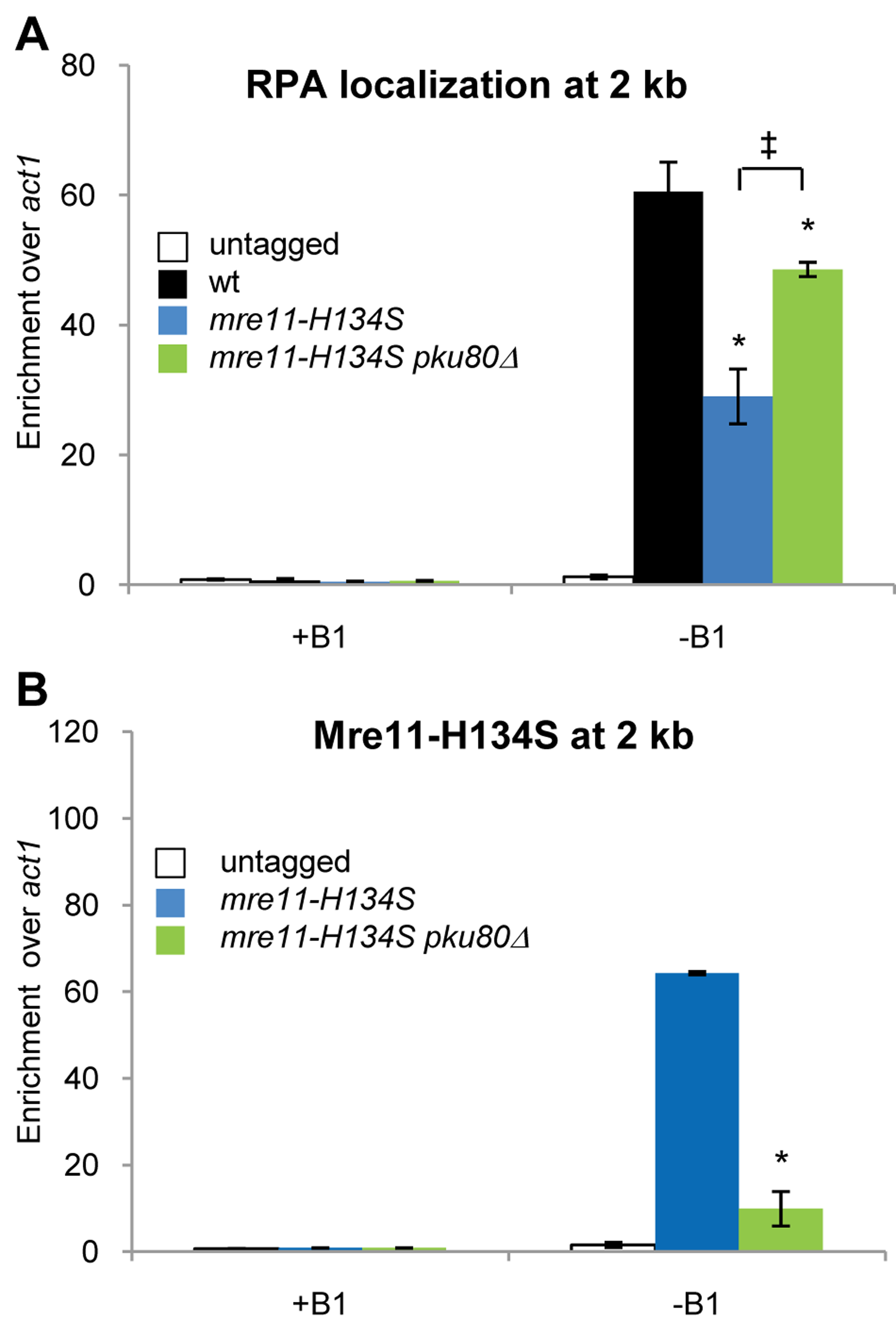

Supplement: Figure S4 — Ku-dependent stabilization of MRN binding decreases RPA localization 2 kb from the DNA end. A. ChIP analysis of RPA (rad11-TAP) shows that RPA enrichment 2 kb from the HO endonuclease induced DSB in mre11-H134S cells is decreased compared to wild type. Deletion of Ku significantly, but partially restores RPA localization compared to wild type. B. Enrichment of Mre11-H134S 2 kb from the HO endonuclease induced DSB in mre11-H134S cells can be reduced by deletion of Pku80. Average and standard deviation (error bar) of three independent experiments are shown. Asterisk depicts statistically significant differences with wild type (A) or the mre11-H134S mutant (B) as determined by a two-tailed Student T-test, p-value≤0.05. (TIF) [file pgen.1002271.s004.tif]

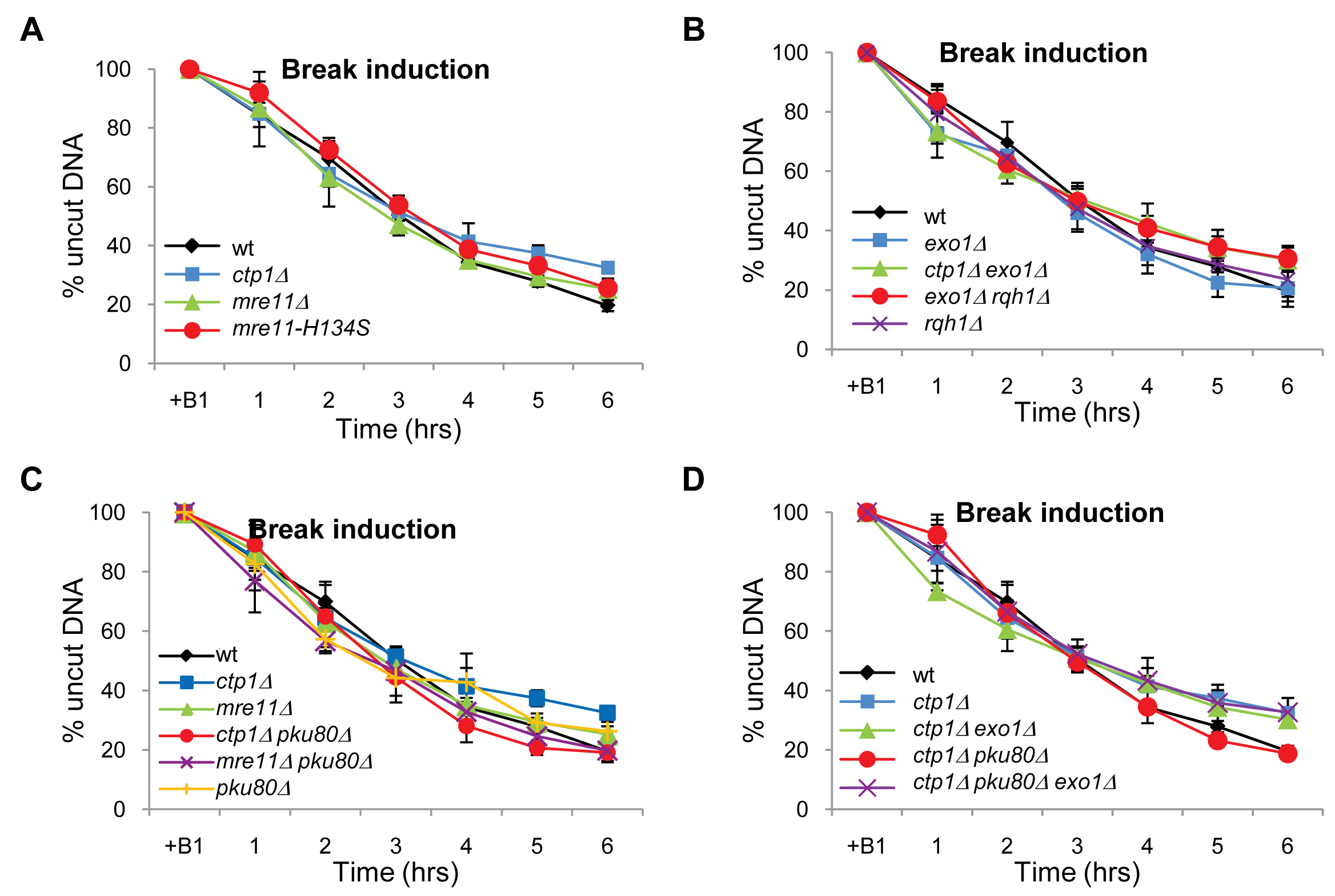

Supplement: Figure S5 — Overlay of break inductions of all strains used. The HO endonuclease is induced at different times after removal of B1 depending on the strain used, sicker strains can take up to 5 hours longer to induce than healthier strains. To directly compare the resection between the different strains, we shift the time course of each strain, to form the most perfect overlay of HO induction as measured by the percentage of uncut DNA. “+B1” represents the repressed condition (HO endonuclease off), time point 1 is the first time point at which the percentage of uncut DNA is lower than 95%. Induction of the HO endonuclease is shown for all strains. (TIF) [file pgen.1002271.s005.tif]

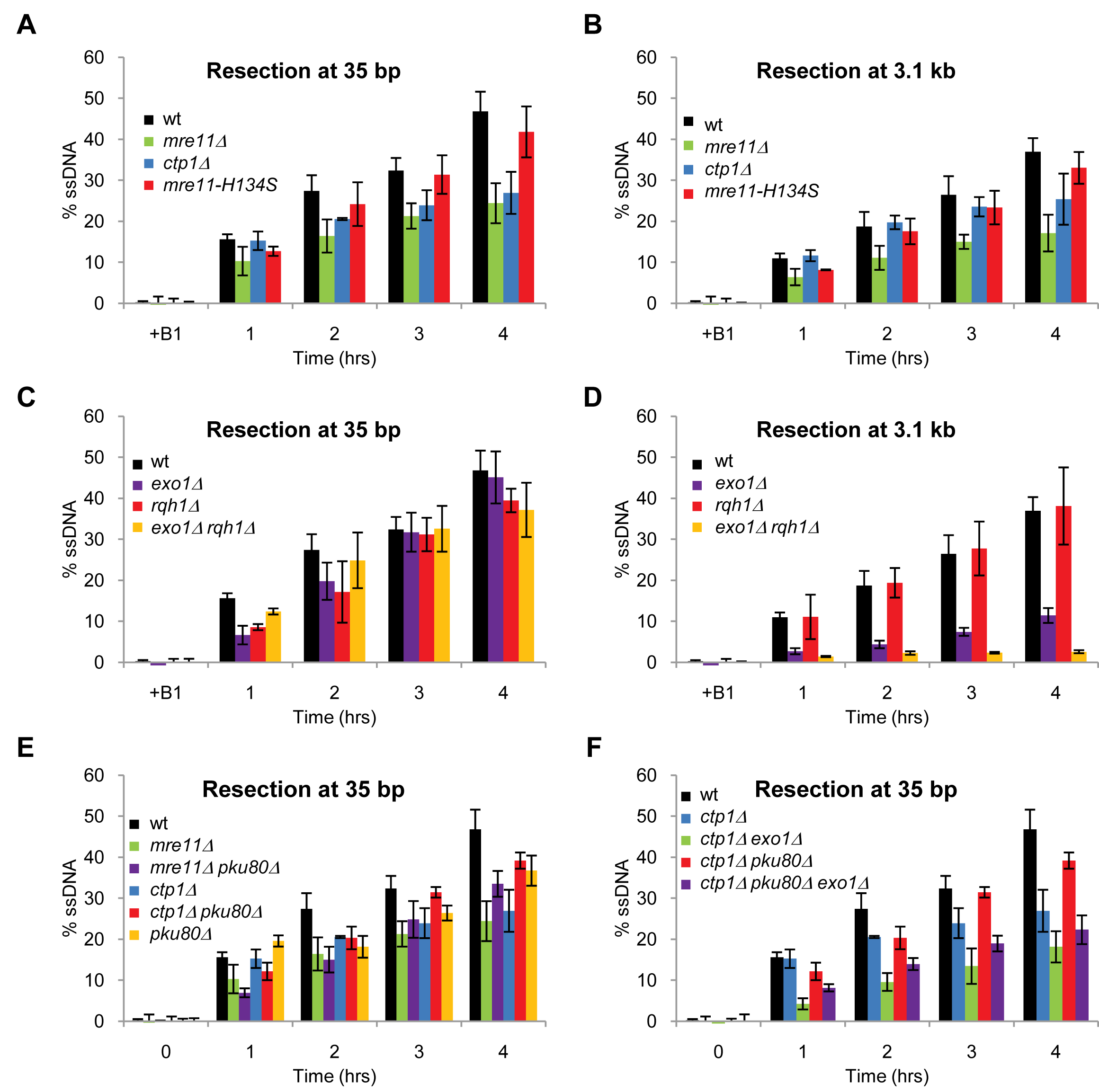

Supplement: Figure S6 — Kinetics study for resection. “+B1” shows the percentage of ssDNA immediately before removal of thiamine. Resection is followed for four hours (until the maximum of cut DNA is reached) from the time point that the percentage of ssDNA in wild type reaches 10%. Averages and standard deviation (error bar) of at least three independent experiments are shown. (TIF) [file pgen.1002271.s006.tif]
