# Supplementary material for: Release of Ku and MRN from DNA Ends by Mre11 Nuclease Activity and Ctp1 Is Required for Homologous Recombination Repair of Double-Strand Breaks
Source: PLoS Genet. 2011 Sep 8;7(9):e1002271. doi: 10.1371/journal.pgen.1002271 (PMC3169521; doi:10.1371/journal.pgen.1002271)
Supplement: Table S1 — Schizo. pombe strains used in this study. (DOC) [file pgen.1002271.s007.doc]

| Strain | Genotype | Source or reference |
| --- | --- | --- |
| PR109 | *h- leu1-32 ura4-D18* | Lab stock |
| PR110 | *h+ leu1-32 ura4-D18* | Lab stock |
| OL229 | *h- leu1-32 ura4-D18 ctp1::natMX6* | This study |
| OL196 | *h- leu1-32 ura4-D18 (or 294) ctp1::natMX6 pku80::hphMX6* | This study |
| OL4177 | *h- leu1-32 ura4-D18 ade6-M210 his3-D1 mre11:kanMX6* | (46) |
| OL4178 | *h- leu1-32 ura4-D18 (or 294) mre11::kanMX6 pku80::hphMX6* | (46) |
| JW4171 | *h- leu1-32 ura4-D18 mre11-H134S-13myc:kanMX6* | (46) |
| OL4172 | *h- leu1-32 ura4-D18 (or 294) mre11-H134S-13myc:kanMX4 pku80::hphMX6* | (46) |
| SC4083 | *h- leu1-32 ura4-D18 (or 294) pku80::hphMX6* | (5) |
| OL4122 | *h+ leu1-32 ura4-D18 ctp1::kanMX6* | (19) |
| PL219 | *h+ leu1-32 ura4-D18 ctp1::kanMX6 pku70::kanMX4* | This study |
| PL235 | *h+ leu1-32 ura4-D18 (or 294) ctp1::kanMX6 pku80::hphMX6* | This study |
| PL224 | *h+ leu1-32 ura4-D18 ctp1::kanMX6 lig4::kanMX6* | This study |
| PL212 | *h+ leu1-32 ura4-D18 ctp1::kanMX6 xlf1::hphMX6* | This study |
| SC4081 | *h+ leu1-32 ura4-D18 xlf1::hphMX6* | (5) |
| TMN3001 | *h+ leu1-32 ura4-D18 pku70::kanMX4* | This study |
| PS2817 | *h+ leu1-32 ura4-D18 ade6-210 his3-D1 lig4::kanMX4* | (4) |
| YYY4403 | *h90 leu1-32 ura4-D18 mat2,3::leu2 mat1-P* | (70) |
| YYY4138 | *h+ leu1-32 ura4-D18 his3-D1 arg3::HOsite(kanMX6) ars1::nmt-HO endonuclease:ampR:his3+:ars1* | (19) |
| YYY4374 | *h+ leu1-32 ura4-D18 his3-D1 ctp1::natMX6 arg3::HOsite(kanMX6) ars1::nmt-HO endonuclease:ampR:his3+:ars1* | This study |
| OL435 | *h+ leu-32 ura4-D18 mre11::natMX6 natMX6 arg3::HOsite(kanMX6) ars1::nmt-HO endonuclease:ampR:his3+:ars1* | This study |
| YYY4359 | *h+ leu1-32 ura4-D18 his3-D1 mre11-H134S-13myc:kanMX6 arg3::HOsite(kanMX6) ars1::nmt-HO endonuclease:ampR:his3+:ars1* | This study |
| PL256 | *h+ leu1-32 arg3::HOsite(kanMX6) ars1::nmt-HO endonuclease:ampR:his3+:ars1* | This study |
| PL250 | *h+ leu1-32 ura4-D18 exo1::ura4 arg3::HOsite(kanMX6) ars1::nmt-HO endonuclease:ampR:his3+:ars1* | This study |
| PL194 | *h+ leu1-32 ura4-D18 rqh1::ura4 arg3::HOsite(kanMX6) ars1::nmt-HO endonuclease:ampR:his3+:ars1* | This study |
| PL204 | *h+ leu1-32 ura4-D18 exo1::ura4 rqh1::ura 4 arg3::HOsite(kanMX6) ars1::nmt-HO endonuclease:ampR:his3+:ars1* | This study |
| PL197 | *h+ leu1-32 ura4-D18 ctp1::natMX6 exo1::ura4 arg3::HOsite(kanMX6) ars1::nmt-HO endonuclease:ampR:his3+:ars1* | This study |
| PL252 | *h+ leu1-32 ura4-D18 (or 294) ctp1::natMX6 pku80::hphMX6 arg3::HOsite(kanMX6) ars1::nmt-HO endonuclease:ampR:his3+:ars1* | This study |
| OL806 | *h+ leu1-32 ura4-D18 (or 294) pku80::hphMX6 arg3::HOsite(kanMX6) ars1::nmt-HO endonuclease:ampR:his3+:ars1* | This study |
| PL329 | *h+ leu1-32 ura4-D18 (or 294) mre11::natMX6 pku80::hphMX6 arg3::HOsite(kanMX6) ars1::nmt-HO endonuclease:ampR:his3+:ars1* | This study |
| PL60 | *h+ leu1-32 ura4-D18 (or 294) ctp1::natMX6 pku80::hphMX6 exo1::ura4 arg3::HOsite(kanMX6) ars1::nmt-HO endonuclease:ampR:his3+:ars1* | This study |
| YYY4140 | *h- leu1-32 ura4-D18 his3-D1 rad11-TAP:kanMX6 arg3::HOsite(kanMX6) ars1::nmt-HO endonuclease:ampR:his3+:ars1* | (19) |
| YYY4141 | *h-* *leu1-32 ura4-D18 his3-D1 rad11-TAP:kanMX6 mre11::natMX6 arg3::HOsite(kanMX6) ars1::nmt-HO endonuclease:ampR:his3+:ars1* | (19) |
| YYY4142 | *h- leu1-32 ura4-D18 rad11-TAP:kanMX6 ctp1::natMX6 arg3::HOsite(kanMX6) ars1::nmt-HO endonuclease:ampR:his3+:ars1* | (19) |
| YYY4455 | *h- leu1-32 ura4-D18 his3-D1 rad11-TAP:kanMX6 mre11-H134S-13myc:kanMX6 arg3::HOsite(kanMX6) ars1::nmt-HO endonuclease:ampR:his3+:ars1* | This study |
| PL369 | *h- leu1-32 ura4-D18 (or 294) his3-D1 rad11-TAP:kanMX6 mre11-H134S-13myc:kanMX6 pku80::hph arg3::HOsite(kanMX6) ars1::nmt-HO endonuclease:ampR:his3+:ars1* | This study |
| PL373 | *h- leu1-32 ura4-D18 (or 294) his3-D1 rad11-TAP:kanMX6 pku80::hph arg3::HOsite(kanMX6) ars1::nmt-HO endonuclease:ampR:his3+:ars1* | This study |
| PL304 | *h+ leu1-32 ura4-D18 (or 294) rad11-TAP:kanMX6 ctp1::natMX6 pku80::hphMX6 arg3::HOsite(kanMX6) ars1::nmt-HO endonuclease:ampR:his3+:ars1* | This study |
| PL325 | *h- leu1-32 ura4-D18 (or 294) rad11-TAP:kanMX6 mre11::natMX6 pku80::hphMX6 arg3::HOsite(kanMX6) ars1::nmt-HO endonuclease:ampR:his3+:ars1* | This study |
| PL258 | *h+ leu1-32 ura4-D18 pku70:3HA:kanMX6 arg3::HOsite(kanMX6) ars1::nmt-HO endonuclease:ampR:his3+:ars1* | This study |
| PL259 | *h+ leu1-32 ura4-D18 pku70:3HA:kanMX6 ctp1::natMX6 arg3::HOsite(kanMX6) ars1::nmt-HO endonuclease:ampR:his3+:ars1* | This study |
| PL332 | *h+ leu1-32 ura4-D18 pku70:3HA:kanMX6 mre11::natMX6 arg3::HOsite(kanMX6) ars1::nmt-HO endonuclease:ampR:his3+:ars1* | This study |
| PL351 | *h+ leu1-32 ura4-D18 pku70:3HA:kanMX6 mre11-H134S-13myc:kanMX6 arg3::HOsite(kanMX6) ars1::nmt-HO endonuclease:ampR:his3+:ars1* | This study |
| OL1551 | *h- leu1-32 ura4-D18 rqh1::ura4 exo1::ura4* | This study |
| OL1552 | *h+ leu1-32 ura4-D18 rqh1::ura4 exo1::ura4* | This study |
| OL4180 | *h- leu1-32 ura4-D18 (or 294) mre11::kanMX6 exo1::ura4* | (46) |
| OL4132 | *h+ leu1-32 ura4-D18 (or 294) ctp1::natMX6 exo1::ura4* | (19) |
| OL4174 | *h+ leu1-32 ura4-D18 (or 294) mre11-H134S-13myc:kanMX6 exo1::ura4* | (46) |
| OL136 | *h- leu1-32 ura4-D18 ctp1::natMX6 mre11::kanMX6* | This study |
| PL294 | *h- leu1-32 ura4-D18 mre11::kanMX6 ctp1::natMX6 pku80::hph* | This study |
| PL88 | *h- leu1-32 ura4-D18 mre11::kanMX6 ctp1::natMX6 pku80::hph exo1::ura4* | This study |
| LLD3427 | *h- leu1-32 ura4-D18 chk1-9myc2HA6his:ura4+* | (19) |
| OL4125 | *h- leu1-32 ura4-D18 chk1-9myc2HA6his:ura4+ ctp1::natMX6* | (19) |
| OL4874 | *h- leu1-32 ura4-D18 chk1-9myc2HA6his:ura4+ mre11-H134S-13myc:kanMX* | (57) |
| OL5001 | *h- leu1-32 ura4-D18 chk1-9myc2HA6his:ura4+ mre11-H134S-13myc:kanMX ctp1::natMX6* | This study |
