# Supplementary material for: Release of Ku and MRN from DNA Ends by Mre11 Nuclease Activity and Ctp1 Is Required for Homologous Recombination Repair of Double-Strand Breaks
Source: PLoS Genet. 2011 Sep 8;7(9):e1002271. doi: 10.1371/journal.pgen.1002271 (PMC3169521; doi:10.1371/journal.pgen.1002271)
Supplement: Table S2 — Primers used in this study. (DOC) [file pgen.1002271.s008.doc]

| Name | Sequence |
| --- | --- |
| 1A | 5’ CGTCCTTCCGATGTTGCTTTAACGCATACTC |
| 1B | 5’ AGACGTATTTGAGTGATAGTGCTCGCTGC |
| 2A | 5’ ATACGACTCACTATAGGGCGAATTGGGTAC |
| 2B | 5' GTCAAGGAGGGTATTCTGGGCCTCCATG |
| 3A | 5’-TGTTGCGGAAAGCTGAAAGGTACCTG |
| 3B | 5’ CTCGCAGTCTGAGAGAGAACTAGATATCGG |
| 4A | 5’ CATAAGGTTTGCATACACCGTTGGGTAGG |
| 4B | 5’ CGGAAAGAACTTGATTGGATTGATTAACACTCATCC |
| 5A | 5' CGTACTAGCTTGTTTGCAACTGAACTCTAGTG |
| 5B | 5' CCGATATCTAGTTCTCTCTCAGACTGCGAG |
| 6A | 5' tagcaccggctcgtctattt |
| 6B | 5' AAGCAATGGGACTTCAATCG |
| 7A | 5' TCAAAGCTGCGAAACAACAC |
| 7B | 5' TCGGTGCAGACGATCAATAA |
