# Supplementary material for: Release of Ku and MRN from DNA Ends by Mre11 Nuclease Activity and Ctp1 Is Required for Homologous Recombination Repair of Double-Strand Breaks
Source: PLoS Genet. 2011 Sep 8;7(9):e1002271. doi: 10.1371/journal.pgen.1002271 (PMC3169521; doi:10.1371/journal.pgen.1002271)
Supplement: Table S3 — Range of the percentage of uncut DNA for each strain at “−B1” as used in Figure 2. (DOC) [file pgen.1002271.s009.doc]

|  | **Average** | **SD** | **Range** |
| --- | --- | --- | --- |
| wild type | 19.5 | 1.9 | 17.3 – 21.8 |
| *ctp1Δ* | 32.5 | 1.0 | 31.5 – 33.5 |
| *mre11Δ* | 25.2 | 2.7 | 22.4 – 27.8 |
| *mre11-H134S* | 25.5 | 3.3 | 23.3 – 29.2 |
| *exo1Δ* | 20.6 | 6.3 | 15.7 – 29.3 |
| *ctp1Δ exo1Δ* | 30.2 | 4.1 | 26.2 – 34.4 |
| *exo1Δ rqh1Δ* | 30.6 | 4.3 | 27.0 – 35.4 |
| *rqh1Δ* | 22.4 | 6.1 | 17.0 31.9 |
| *ctp1Δ pku80Δ* | 18.8 | 2.2 | 17.1 – 21.3 |
| *mre11Δ pku80Δ* | 19.8 | 3.5 | 16.5 – 24.2 |
| *pku80Δ* | 26.3 | 3.1 | 22.7 – 28.1 |
| *ctp1Δ pku80Δ exo1Δ* | 32.7 | 4.8 | 28.1 – 37.7 |
